# Incremental data integration for tracking genotype-disease associations

**DOI:** 10.1371/journal.pcbi.1007586

**Published:** 2020-01-27

**Authors:** Tomasz Konopka, Damian Smedley

**Affiliations:** William Harvey Research Institute, Queen Mary University of London, London, United Kingdom; University of Chicago, UNITED STATES

## Abstract

Functional annotation of genes remains a challenge in fundamental biology and is a limiting factor for translational medicine. Computational approaches have been developed to process heterogeneous data into meaningful metrics, but often do not address how findings might be updated when new evidence comes to light. To address this challenge, we describe requirements for a framework for incremental data integration and propose an implementation based on phenotype ontologies and Bayesian probability updates. We apply the framework to quantify similarities between gene annotations and disease profiles. Within this scope, we categorize human diseases according to how well they can be recapitulated by animal models and quantify similarities between human diseases and mouse models produced by the International Mouse Phenotyping Consortium. The flexibility of the approach allows us to incorporate negative phenotypic data to better prioritize candidate genes, and to stratify disease mapping using sex-dependent phenotypes. All our association scores can be updated and we exploit this feature to showcase integration with curated annotations from high-precision assays. Incremental integration is thus a suitable framework for tracking functional annotations and linking to complex human pathology.

## Author summary

Human diseases are often caused or influenced by genetic factors. The link between a particular gene and a specific disease is well-established in some cases. However, the roles of many genes are still unclear and many diseases do not have an understood genetic mechanism. Dissecting such interactions requires using a range of experimental approaches and assessing the results in a holistic manner. Computational methods already exist for comparing phenotypes observed in models and patients, and they work well when the phenotypes are detailed. In this work we argue that algorithms should also be able to report meaningful assessments based on preliminary data, and to update reports in a coherent manner when new information comes to light. These requirements lead to specific mathematical properties, which define incremental integration. We implement these requirements in a computational framework. We study the extent individual rare human diseases might be recapitulated by animal models. We compute gene-disease associations using data from public resources, including previously unused negative data. Altogether, these examples illustrate the framework can use observations in model systems to track gene-disease associations in the human context.

## Introduction

Technological advances ensure that a growing range of assays are available to probe biological systems, from profiling of single cells to phenotyping of entire organisms. These data open possibilities to study the role of genes in fundamental biological processes as well as disease. However, a complete understanding of gene function is only achieved by synthesizing several lines of evidence [1,2]. Some aspects of this data-integration task are addressed by specialized approaches that, for example, perform analyses of multi-omic data [3], meta-analyze related cohorts [4], or summarize outputs of distinct computational approaches [5]. Such studies prioritize associations and some provide factorizations that can lead to conceptual insights. Nonetheless, it is often difficult to interpret how results might change in the presence of new information, especially from a distinct type of assay. Furthermore, integration approaches that are reasonable for isolated analyses can include features that are unsuitable when evidence is collected over an extended period. Thus, methods are needed that are compatible with a wide range of experimental workflows and that can capture an evolving state of knowledge.

As an illustration of the challenges involved in data integration, consider two hypothetical experiments that both provide strong evidence for a certain effect, for example, a gene-phenotype association (S1 Fig). A summary of this data should be expected to provide strong support for the effect. Indeed, approaches based on averaging or consensus would report this outcome. However, consider recording an additional experiment that provides consistent, but weaker, evidence. An integration strategy based on averaging would report a weaker association than at first. This is appropriate when estimating effect size, but it is counter-intuitive when the aim is to determine whether the effect is true or not. An alternative strategy based on consensus would find that the new evidence does not alter the most common observed result and would report the same association as before. This discards valuable new information. Issues are compounded when the pool of data grows further. Averaging-based strategies allow an association to appear weaker despite growing supporting evidence, and consensus-based approaches report unchanged results in some cases and shift in a drastic manner when one experimental outcome outcompetes another. Indeed, such effects occur in practical integration efforts involving heterogeneous components[6–8].

The challenges in data integration are not limited to those arising from averaging and consensus. Normalization can have a similar impact on the interpretability of association scores. In approaches that assess many associations at once, for example in the context of gene networks, normalization can suggest that adding data for one component can impact scores of unrelated components. This is appropriate when the goal is to rank associations, but not when the aim is to assess whether an association is present. Other complications arise when data originate from a variety of sources and assay types. Approaches designed for high-throughput (omic) data might not be able to incorporate evidence from low-throughput experiments, even when these validate findings [4]. Meta-analysis of raw datasets, while alleviating some of the challenges and providing statistical rigor, often cannot cope with data of different modalities.

Despite these difficulties, there is a need to assess heterogeneous collections of data to track gene function and to understand their impact on human disease. Curation teams currently use manual systems for this purpose [9–11]. Because the volume and richness of data is increasing, however, there is a need for a computational framework that can incrementally build confidence in associations. We suggest that such a framework should satisfy a number of criteria to maintain consistency and coherence. Incremental integration should score an association higher whenever new evidence confirms an initial finding, maintain an association at a constant level when new data are not relevant or pertain to a different domain, and decrease an association in cases of conflict. The framework should be adaptable to a wide range of settings and inputs, including high-throughput and low-throughput assays. Computational efficiency is also desirable if associations are to be updated with ease.

A practical application for incremental integration is tracking animal models and their associations with disease. The International Mouse Phenotyping Consortium (IMPC) is knocking-out individual genes in mice, measuring resulting changes in animal phenotypes, and assessing the dataset with a standardized statistical pipeline [12]. The project has produced animal models that phenocopy human genetic diseases [13] and identified genotype-phenotype associations in body development [14], eye development [15], hearing [16], and metabolism [17]. Complementary data are also generated in bespoke experiments by individual research laboratories and collected in the Mouse Genome Database [18]. The continual growth of these repositories has prompted the evaluation of new techniques to determine the statistical significance of individual phenotypes [19]. From the perspective of clinical translation, it is also important to track how evolving annotations modulate connections to human disease, but this has not been addressed so far.

Here we describe a framework for data integration that is consistent with the requirements of incremental updates and is suitable to track model-disease associations. We first describe a record-keeping approach that combines distinct experimental measurements into a single value per phenotype. Then, we use inter-phenotypic relationships encoded by ontologies to perform comparisons with disease annotations. Our approach uses a mathematical transformation that stems from Bayes’ formula, but is adapted to the context of phenotyping. Importantly, our approach can incorporate positive as well as negative evidence, accommodate high-throughput as well as precision experiments, and update scores with new evidence.

## Results

### Update-driven integration

Incremental data integration is the task of tracking and updating an association score, for example between a genotype and a phenotype, in the light of experimental evidence. A relevant mathematical transformation for this task stems from Bayes’ formula, which takes an existing probability as input and returns an adjusted value (Methods). This can be used in a direct manner to integrate measurements pertaining to an individual phenotype. The calculation begins with a prior probability for the phenotype and updates it with a strength that depends only on the statistical power and the false positive rate of the measurement assay. Because such properties can be estimated for any experimental assay, the approach is compatible with disparate data sources and gives appropriate weight to each input. Thus, measurements from a pilot can be used alongside a well-powered study, with the two sources weighted appropriately.

The Bayes transformation has mathematical properties that translate into operational benefits. First, the transformation defines a commutative group. Several transformations can be composed together and the group properties guarantee that a score always increases with supportive evidence and that the final outcome does not depend on the order of operations. It is also possible to integrate contradictory evidence to decrease a score (S2A Fig). Thus, the transformation satisfies the primary requirements for incremental integration. Second, the transformation always yields output in the unit interval, [0,1]. This provides intuition as a measure of confidence. It also implies that an integrative score can achieve a maximal value regardless of whether individual pieces of evidence are strong or weak (S2B Fig).

### Scoring with ontology-based phenotype profiles

While the application of the Bayes transformation is straightforward for single features, animal models as well as human diseases are characterized by several phenotypes. Additional design choices are required to compare them in a systematic manner. To this end, we first defined how data for individual phenotypes should be incorporated into holistic profiles. We then implemented an algorithm to quantify how well one holistic profile matches another. Both steps drew on phenotype ontologies.

Ontologies are acyclic graphs with nodes, called terms or classes, connected by links, which are hierarchical relations. Ontologies are community-maintained and systematize definitions of features that are relevant to a subject domain. They are thus a solid base on which to build a framework for data integration. In this work, we use phenotypes defined by the Mammalian Phenotype (MP) ontology because these are relevant to both humans and mice [20]. The systematic definition of ontology terms also enables us to estimate the frequency of each mammalian phenotype, thus providing background values (priors) that are the starting points for Bayes transformations (Methods).

**Fig 1:**
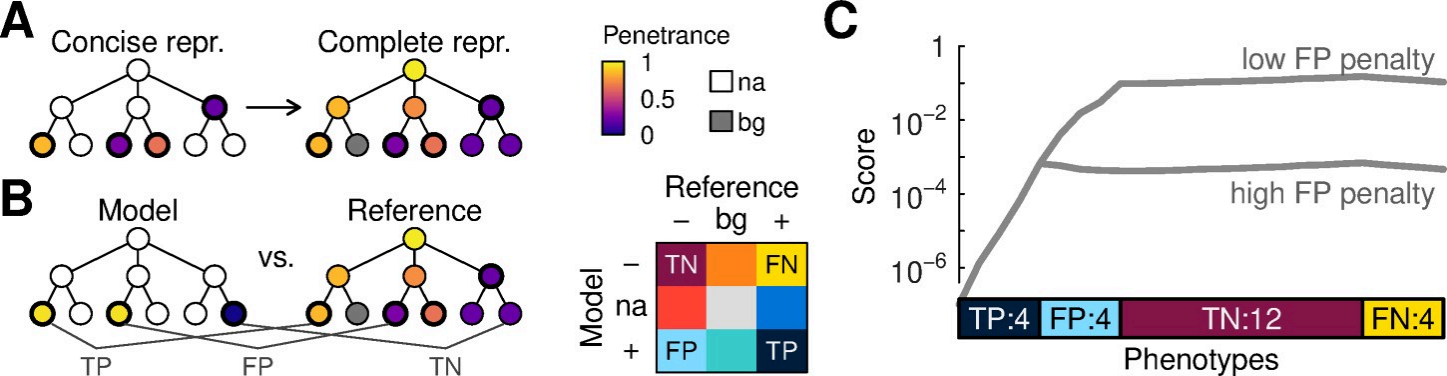
Scoring phenotypic similarities. (A) Schematic of a concise representation (repr.), with data for many phenotypes not available (na), and a complete representation, with values for all phenotypes. Graphs represent an ontology hierarchy. Colors indicate the strength of a phenotype, e.g. penetrance of a disease phenotype. (B) Scoring of a model against a reference proceeds one phenotype at a time. Each comparison produces one of nine possible outcomes depending on the values assigned to the model, reference, and the background (bg) level of each phenotype. (C) Incremental score integration wherein a score begins at a low level (left) and is adjusted as evidence becomes available. Phenotype evidence is shown grouped by true positive (TP), false positive (FP), true negative (TN), and false negative (FN) status. Lines display two integration paths that depend on how false-positives are handled.

We defined two types of representations for holistic phenotype profiles (Fig 1A). The first representation, which we call ‘concise’, contains data only for those phenotypes that have been measured in the laboratory. In this representation, most features defined by the ontology are not annotated. The second representation, which we call ‘complete’, is inferred from the concise data using the ontology graph. It contains explicit values for all terms in the ontology and it is convenient for looking up values for arbitrary phenotypes, but it obfuscates the distinction between measured and inferred phenotypes. Overall, the ‘concise’ and ‘complete’ representations hold similar information, but with different trade-offs to suit downstream calculations.

Assessing the concordance of two phenotypic profiles is a nontrivial task and several approaches have been previously described, including proposals akin to computing distances between complete representations [21,22]. Here, we defined an algorithm motivated by the properties of the Bayes transformation. Given a model and reference profile, our algorithm begins with a small score for the association between the two. It is tempting to interpret it as an *a-priori* probability that the model phenocopies the reference. This provides intuition, but our approach is not calibrated to preserve this interpretation throughout the scoring process; the prior and the subsequent probabilities are thus arbitrary values from which we can track upward and downward updates.

The algorithm compares phenotype values in the model concise representation with corresponding values in the reference and the background (Fig 1B). The reference value can be lower, equal to, or greater than the phenotype background rate, and the same possibilities exist for the model. There are thus nine scenarios for a score update (Fig 1B inset). Four of these are familiar from conventional machine learning: true positives (TP) when both the model and the reference have values greater than the background; true negatives (TN) when both the model and the reference are lower; false positives (FP) when the model is greater but the reference is lower; and false negatives (FN) when the model is lower but the reference is higher. The five other scenarios (expected positives and negatives, anticipated positives and negatives, and undetermined) occur when the reference or the model have values exactly equal to the background.

Each scenario leads to a corresponding score update (Methods). A true positive always produces a confirmatory update, but its strength is modulated by the value of the model, reference, as well as the background probability. Thus, the update is strong when the model is well-measured, when the phenotype has high penetrance, and when the phenotype is rare in a background population. Scenarios with negative data (FN, TN) are handled in a similar manner. Scenarios in which one of the values is equal to the prior do not lead to any adjustments.

Comparisons that lead to a false positive (FP) outcome, being contradictions between model and reference, should be expected to lead to a penalty. However, when a reference is not annotated with a certain phenotype but has a more general feature in the ontology hierarchy, it may be desirable to increase the score of association to capture the fuzzy similarity. Thus, we implemented the update for FP phenotypes as a composition of confirmatory and contradictory components. The balance between these components cannot be determined from first principles and is a free parameter (Fig 1C).

### Assessment of disease annotations

We began exploring applications for our scoring framework by studying disease annotations. Diseases are characterized by several symptoms and phenotypes and we wished to summarize to what extent these features can be recapitulated by our representations, and whether existing annotations can discriminate between them.

Disease features and their prevalences, both encoded as terms from the Human Phenotype (HP) Ontology, were extracted for 3,331 diseases defined by ORPHANET[23]. Annotations in this dataset vary greatly in extent, with the number of concise HP terms ranging from 1 to 176 per disease (mean, 20). To later compare these descriptions to mouse models, we mapped individual HP features into analogs in the Mammalian Phenotype (MP) ontology using an ontology comparison algorithm [24] (S1 Table). We then defined a set of complete disease representations (i.e. representations in which all phenotypes have values, either from direct annotations or via ontology-based inference). After this step, many phenotypes retained values equal to priors. It is unclear whether they represent features that are not manifest in a disease or whether their status is unknown. We thus adjusted their values by a multiplicative factor, thereby making a compromise between the two interpretations.

Having obtained representations for diseases that are compatible with our framework, we explored the extent of annotation in the ORPHANET dataset by computing sums of values in the disease representations in both the original HP- and translated MP-space. These sums correlate with each other and with other measures such as information content (S3 and S4 Figs). Similar to the raw annotation counts, the sums span a wide numeric range. Some of the heterogeneity can be attributed to disease impact: diseases that affect multiple organ systems are associated with more phenotypes (anova p. 4⨉10^−121^, S5 Fig). However, incomplete curation is also a factor. For example, Phenobarbital Embryopathy (ORPHA:1919) included only one concise phenotype (global developmental delay) in our dataset despite being assigned to six disease classes (swallowing disorders, teratologic disorders, and others). Another example was Isolated Complex 1 Deficiency (ORPHA:2609) which contained a phenotype for a subcellular feature (abnormal mitochondria in muscle tissue) while lacking annotation on its physiological effect.

**Fig 2:**
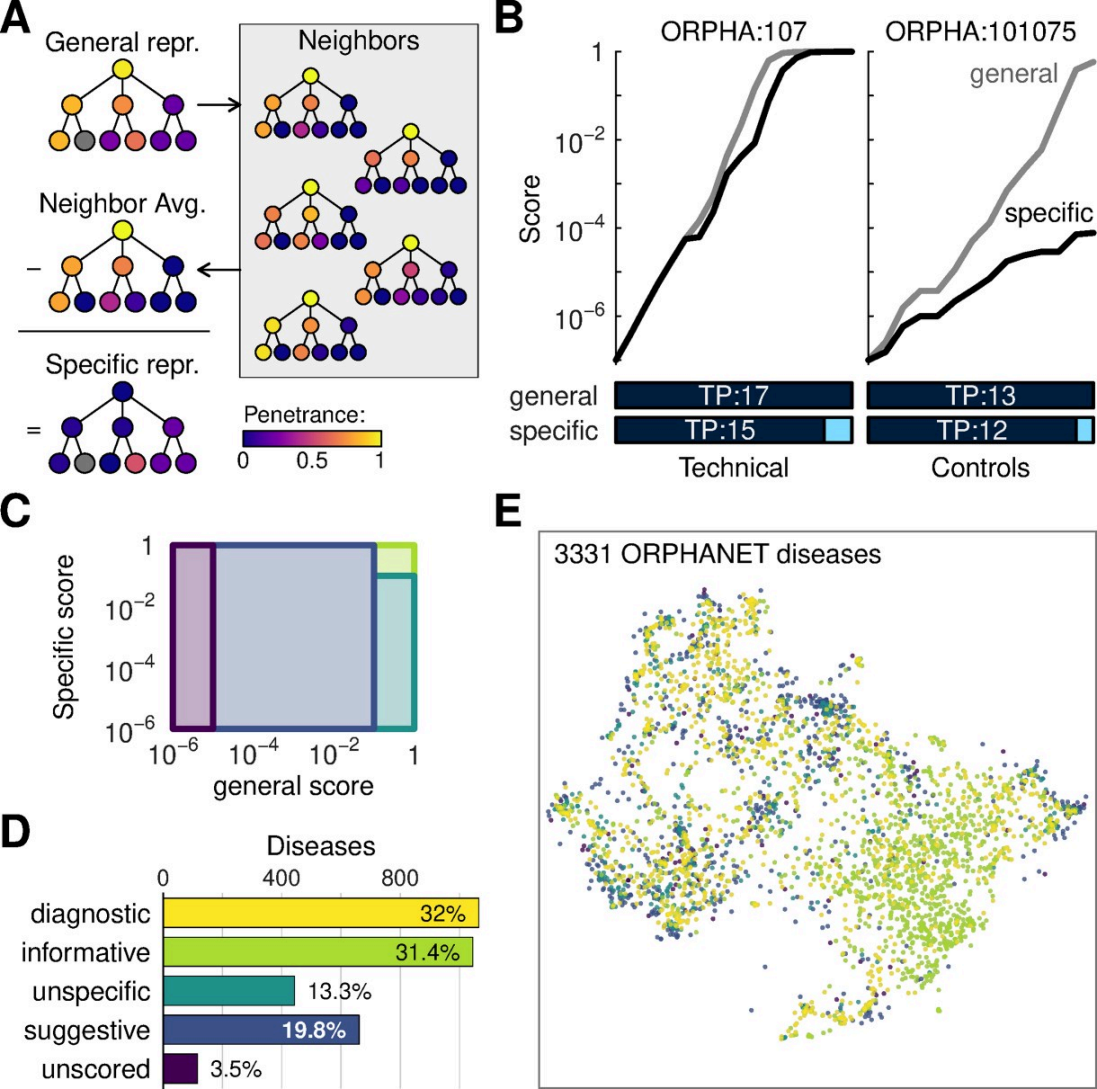
Assessment of disease annotations. (A) Definition of specific disease representation. Starting from a complete representation of a disease, nearest neighbors are identified and averaged; the specific representation is the difference between the original representation and the neighbor average. (B) Examples of scoring technical controls against their source disease. Phenotypes along the horizontal axes are displayed using the same color scheme as in Fig 1C with true positives (TP) followed by false positives (FP). Left panel shows a disease with informative annotations; right panel shows a disease for which the specific score fails to reach saturation. (C) Legend for disease classification based on the technical controls. Rectangles and colors define regions in the score space classified as informative (green), unspecific (light blue), suggestive (dark blue), unscored (purple). (D) Summary of disease classification based on technical controls. (E) Visualization of the disease landscape. Dots correspond to diseases, arranged by similarity of their general phenotypes using UMAP. Colors indicate the annotation class using the color scheme from (C) and (D).

Among the diseases that had extensive annotations, several phenotypes were shared across more than one disease. For example, more than 200 diseases included reports of joint stiffness. We thus set out to define a complementary set of representations to emphasize features that are specific to each disease (Methods). For each disease, we identified five other phenotypically similar ones. The residual between the general disease profile and the average of its nearest-neighbors defined a new profile (Fig 2A). Many phenotypes in these new profiles, especially high-level ontology terms, were assigned values equal or very close to their background level.

Next, we used technical controls to assess the power of phenotypic measurements to phenocopy these profiles. We first created a set of models with synthetic confirmatory measurements for the disease phenotypes. We then scored these controls against all references. In this approach, every disease should be matched to at least one model–its own technical control. Indeed, technical controls often achieved high scores against their source disease, e.g. Bor Syndrome (ORPHA:107) (Fig 2B). However, some technical controls did not achieve high scores due to the small number of annotations present. A class of controls achieved high scores against the general reference profile but not against the specific profile, e.g. X-linked Charcot-Marie tooth disease, type 1 (ORPHA:101075) (Fig 2B). The most drastic cases were due to annotations that, as for Charcot-Marie disease, assign similar phenotypes to several subtypes of a core disease.

To summarize results from the technical controls, we categorized diseases according to their hits (Fig 2C and 2D, S3 Table). Diseases such as ORPHA:107 that scored high with their matched controls comprised a large proportion of the total, 63.4%, so we further partitioned it into two tiers. Diseases were termed ‘diagnostic’ when a technical control achieved a better score against its disease than with any other disease. The naming is here motivated by a hypothetical scenario in which the control could be identified based on its phenotypes. Remaining diseases that had high scores but did not satisfy ‘diagnostic’ criteria were named ‘informative’. Among diseases for which the control did not achieve top scores, disease such as ORPHA:101075, were termed ‘unspecific’ when they scored high according to their general profile but not their specific profile. Cases that did not achieve high scores through either profiles were named ‘suggestive’. Diseases for which the matched controls did not achieve a minimal improvement over the prior were termed ‘unscored’.

To understand these classes in terms of disease physiology, we generated a map of the disease landscape. Although this separated diseases belonging to major physiological areas (S6 Fig), our labels were spread over the entire map (Fig 2E). Thus, the ORPHANET and HP datasets contain well-annotated as well as less-well-annotated entries across the entire disease spectrum.

We repeated similar calculations based on disease profiles and technical controls without the HP-MP translation (S7 Fig). A higher fraction of diseases achieved the ‘diagnostic’ label. Nonetheless, several diseases remained ‘suggestive’ or ‘unspecific’. We also explored the effect of free parameters and found that the penalty associated with FPs primarily affects the distinction between ‘diagnostic’ and ‘informative’ labels (S8 Fig). The number of nearest neighbors used to define specific disease profiles, in contrast, only has slight impact (S9 Fig).

### Assessment of mouse lines as models of human disease

**Fig 3:**
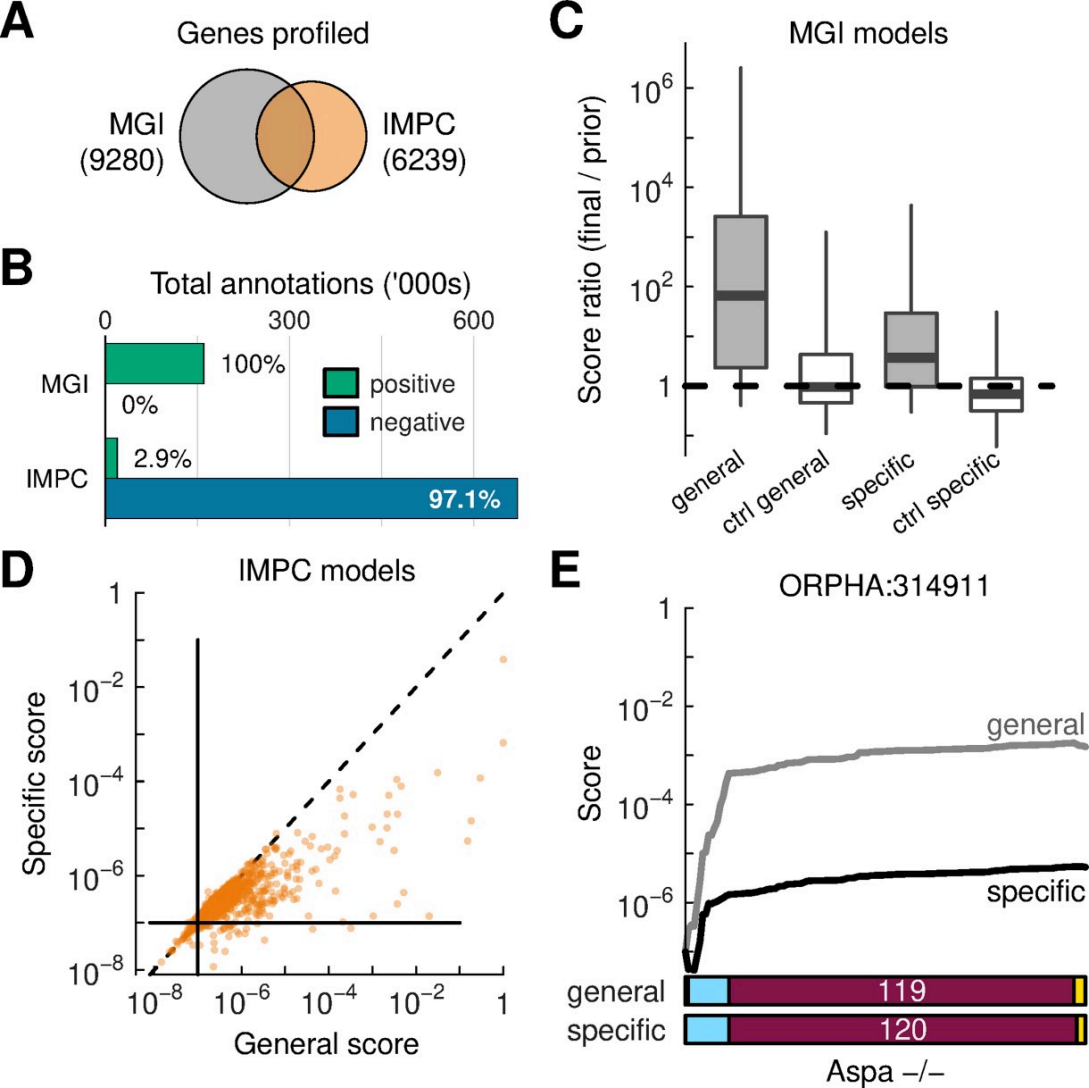
Known disease-gene associations. (A) Overlap of mouse genes in the MGI and IMPC databases. (B) Summary of the number of phenotype annotations in the two resources; positive phenotypes convey that an abnormality is observed in a knock-out animal; negative phenotypes are reports that a measurement was attempted but not called as significant. (C) Comparison of scores for known disease-gene pairs based on MGI data using general and specific disease profiles (gray boxes). Each set of scores is compared against matched, randomly selected disease-gene pairs (controls, white boxes). Box features correspond to distribution medians (center line), interquartile ranges (box borders) and 5%-95% quantiles (whiskers). (D) Comparison of general and specific scores computed using IMPC data. Horizontal and vertical bars indicate score priors. The diagonal line is a visual guide. (E) An example of score integration for a known disease-gene pair using IMPC data.

Having verified that our approach can match phenotype profiles, we turned to animal models for insights about human disease. Annotations for knock-out mouse lines were downloaded from the Mouse Genome Informatics [18] (MGI) and the International Mouse Phenotyping Consortium [25] (IMPC) data portals. Phenotypes were grouped by background strain, knock-out allele, and zygosity to represent individual models. Overall, we obtained data for 30,066 models from the MGI and 8,673 models from the IMPC, describing 9,280 and 6,239 mouse genes, respectively, with 2728 genes in both datasets (Fig 3A).

Although both the IMPC and MGI label phenotypes using the Mammalian Phenotype (MP) ontology, the datasets have structural differences. IMPC data derive from an ongoing phenotypic screen that aims to profile all genes in the mouse genome [12]. In contrast, MGI data summarize bespoke efforts by diverse research groups. Thus, only 2% of genes in the IMPC dataset are described by more than two mouse models, whereas that fraction is 35% for the MGI dataset. Annotations of positive phenotypes for MGI mice are more detailed than for IMPC models, but the latter includes systematic records for performed measurements that were not found to be statistically significant, i.e. negative phenotypes (Fig 3B, S10 Fig). The two resources thus have unique characteristics.

Previous studies have shown that when a mutation in a gene causes a disease, then mouse models with a knock-out in the gene ortholog can phenocopy the disease and that this relationship can be quantified [13]. To reproduce those findings with our framework, we extracted curated disease-gene pairs [23] and scored the relevant MGI models against the disease general and specific profiles. Calculations started with a prior probability of 1⨉10^−7^ and although this level may not be realistic as a probability of incidence for individual diseases, it is a baseline from which to consider increases or decreases. We expected scores between known disease-gene pairs to yield higher values than other pairings selected at random (Methods). This was confirmed for all values of the free parameters in our framework (S11 Fig). From those results, we calibrated parameters so that random pairs show, on average, no enrichment compared to the prior (Fig 3C). The same trends were present using IMPC models (S12 Fig), confirming that data from high-throughput phenotyping can recapitulate characteristics of human diseases.

Given that IMPC data contains both positive and negative phenotypes, we investigated the value of the negative data for disease matching. Adding negative data increased scores for known disease-gene pairings, indicating concordance between the measurements and disease knowledge. However, negative phenotypes also increased scores for randomly-matched controls (S12 Fig). This reflects the fact that most diseases are associated with only a narrow set of phenotypes, so a record for the absence of a specific phenotype in a mouse model enhances a match with most diseases.

Despite the propensity of known disease-gene associations to score higher than random pairs, most final scores were modest in magnitude. This indicates that the scoring approach leaves opportunities to integrate additional data in the future. Scores against specific disease profiles were often lower than against general profiles (Fig 3D). To explore this further, we scrutinized the incremental updates. As an example, we considered a mouse with homozygous knock-out in gene Aspa, coding for an enzyme that catalyzes the production of aspartate, scored against severe Canavan disease (ORPHA:314911), a neurodegenerative disease (Fig 3E). The breakdown revealed that much of the enrichment in the general score was due to FP matches. Those are model phenotypes that are not concordant with the disease, but for which other disease phenotypes are sufficiently similar in the ontology structure to produce a score increase. These FPs did not substantially increase the specific score, suggesting that the upstream phenotypes are present in other diseases.

To compare our scoring approach with existing algorithms for quantifying disease-gene associations, we obtained scores for curated disease-gene pairings from the IMPC data portal. These existing scores are computed using an algorithm [21] with many similarities to our approach, but not designed for incremental updating. While the score sets were correlated, the incremental scoring system avoided assigning high scores to poorly annotated models (S13 Fig). As a result, the probability-based scores correlated better with the levels of disease and model annotation (S14 Fig).

### Ranking of disease-gene associations

**Fig 4:**
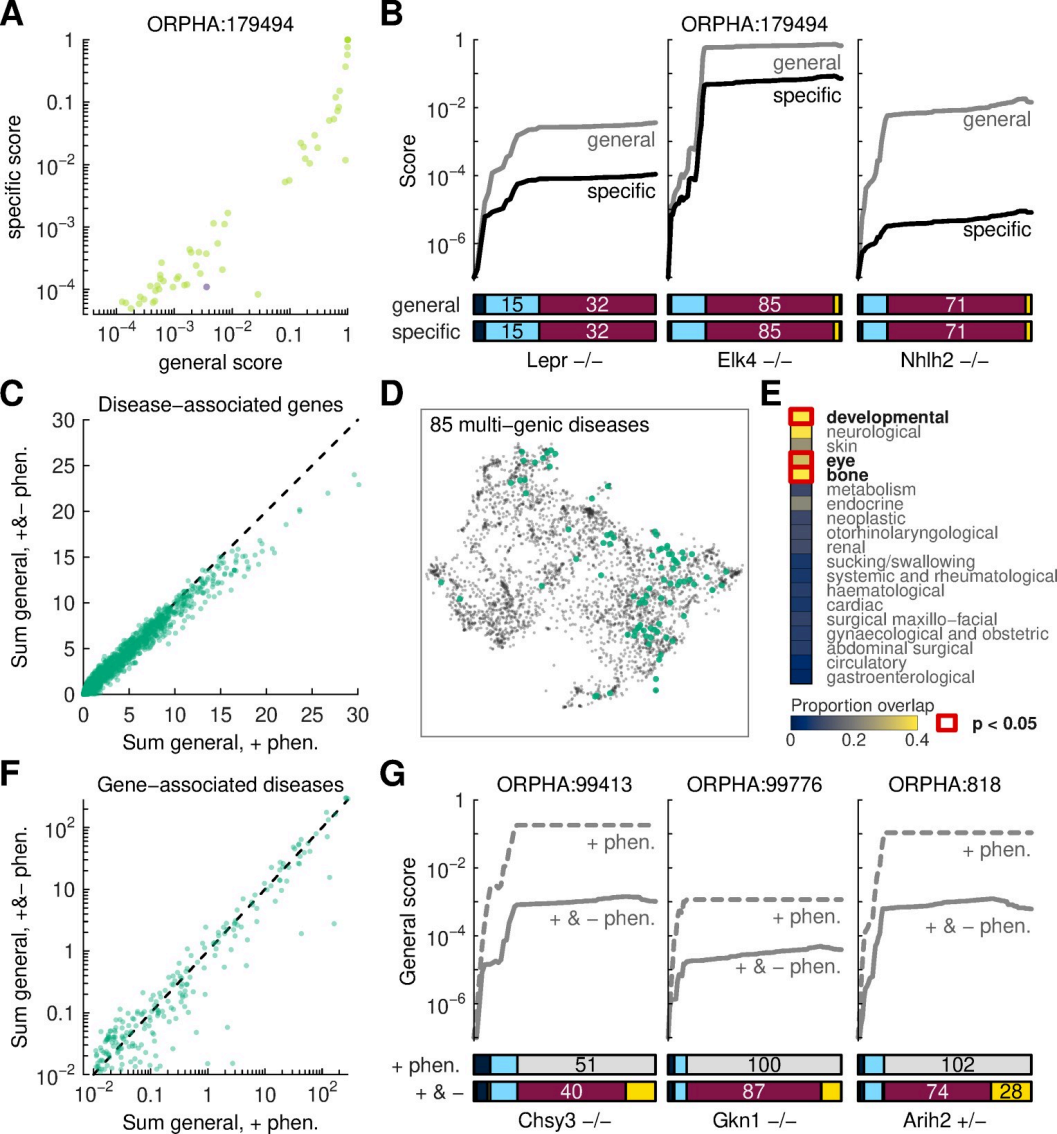
Novel disease-gene associations. (A) Summary of all IMPC models scored agains a single disease; the model with a knock-out in the disease-causative gene is indicated. (B) Details of score calculations. Left: scores for the known disease gene; middle: a novel gene that achieves high general and specific scores; right: a novel gene that only displays unspecific phenotypes. (C) Summary of the number of genes associated to indvidual diseases, computed with and without negative phenotypes. Dots represent diseases. Dots below the diagonal line convey that the number of candidate genes associated with those diseases is lower once negative data is considered. (D) Visualization of disease with multiple gene hits on a semantic similarity map. (E) Comparison of putatitive multi-genic diseases with major disease categories. Overlap is computed via set intersection followed by normalization by the disease category size. Enrichment is computed via Fisher tests. (F) Summary of the number of diseases associated with individual genes, computed with and without negative phenotypes. (G) Examples of score calculations for which general scores computed with negative phenotypes are substantially lower than with positive phenotypes alone. All lines represent scores against general disease profiles. By construction, lines based only on positive phenotypes appear flat during integration of TN and FN data.

A canonical use for similarity scores between disease profiles and mouse models is to identify novel disease-gene associations. To this end we computed scores for all disease and model pairs. As a case study, we investigated models associated with obesity due to Leptin receptor gene deficiency (ORPHA:179494). An IMPC model with homozygous knock-out in the causative gene (Leptin receptor, *Lepr*) produced high scores against the general and specific disease profiles, but several other genes scored high as well (Fig 4A). Given this disease is defined by its molecular cause, i.e. Leptin receptor deficiency, these additional hits cannot be considered as candidates for causation.They may nonetheless offer insights into pathways and molecular regulators. We thus investigated a few genes via score update charts (Fig 4B). The *Lepr* knock-out produced curves with a monotonic, increasing pattern. Curves for another candidate (*Elk4* knock-out) revealed that the gene had been phenotyped by more assays, that some updates decreased scores, and that much of the final scores could be attributed to a small number of false-positive matches. Such patterns can prompt manual investigation: in this case, score decreases were due to weight-loss phenotypes and strong false positives originated from immunophenotyping. These data suggest that if the gene is relevant to the disease, its role must be indirect and involve inhibitory regulation. In another example (*Nhlh2*), scores against the general and specific profiles deviated substantially, again suggesting an indirect role. Overall, the update charts reveal that several genotypes are consistent with the disease and provide hints for downstream analysis and interpretation.

For diseases where the causative genes are not known, an analysis of mouse models can prioritize gene candidates. Creating definitive shortlists of candidates for each disease would require setting thresholds for the general and specific scores. Such thresholds are to a large extent arbitrary and the heterogeneity in disease annotations means that it would be inappropriate to set a universal value. Thus, we opted to summarize each disease from an aggregate perspective. In this direction, we computed sums of model scores for each disease. These sums can be interpreted as estimates for the number of genes that phenocopy each disease. Using IMPC data, sums computed from positive phenotypes correlated with sums computed using positive and negative data (Fig 4C). Importantly, for diseases with the largest sums, the effect of negative phenotypes was to reduce the potential hits by 20–30%. Together with previous observations that negative phenotypes generally increase scores for known (true) disease-gene pairs, this reduction implies that negative phenotypes improve the prioritization of disease-relevant genes. Because phenotypic comparisons can highlight many non-causative genes, the reduction of gene candidates simplifies the search for true disease-gene associations.

Next, we studied the relationship between score sums and the breadth of disease annotations (S15 Fig). From this modeling, we identified diseases associated with more genes than would be expected from the trend. Multi-genic diseases spanned most disease classes and showed slight enrichment in some disease categories (Fig 4D and 4E, S16 Fig). It is unclear whether the enrichment is due to the preferential ability of mouse models to phenocopy those diseases, differential annotation quality, or residual technical effects.

From the opposite perspective, we studied the number of diseases that might be associated with individual genes, i.e. pleiotropy in the disease space. Paralleling our previous approach, we computed sums of all disease-gene scores for a given gene. Sums computed from positive phenotypes and from both positive and negative phenotypes were again correlated (Fig 4F). However, we did not observe a systematic reduction of associations for well-annotated genes. Instead, there were isolated genes for which the number of associated diseases was substantially lower when negative phenotypes were included. Score reductions occurred through moderation of score updates due to TP and FP phenotypes (Fig 4G). This means that negative data adjusted the strength of the positive score updates, effectively using these data to capture phenotype penetrance.

### Data stratification and augmentation

**Fig 5:**
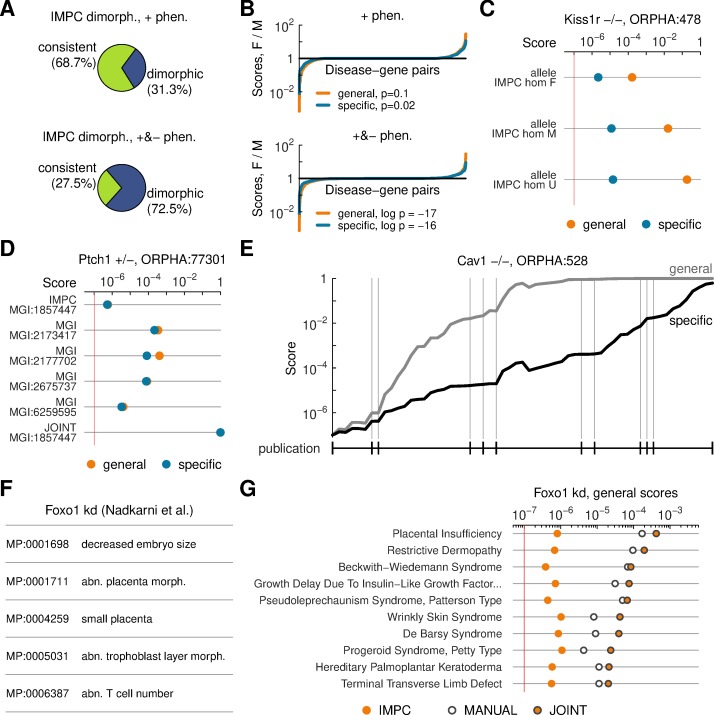
Data integration and stratification. (A) Summary of phenotypic sexual dimorphism in IMPC models. Pies displays proportions of models with dimorphic phenotypes based on positive (+) phenotypes alone (top) and based on both positive and negative (–) phenotypes (bottom). (B) Effect of sexual dimorphism on scoring known disease-gene associations. Vertical axes show ratio of scores as computed from female (F) and male (M) animals. Horizontal axes display ordered disease-gene pairs. Lines distinguish scores computed against general and specific disease profiles. P-values are computed using the binomial test. (C) Example of score comparisons for one disease-gene pair. Rows display female (F), male (M), and sex-unspecified (U) models. (D) Example of a calculation merging MGI and IMPC data for a known disease-gene association. (E) Score calculations for a known gene-disease associations using data from MGI informed by several publications. Phenotypes on the horizontal axis are grouped by publication (notches, vertical guide lines). Lines indicate scores against the general and specific disease profiles. (F) Set of mamallian phenotypes manually extracted from an original research article. (G) Summary of diseases best matched against the phenotypes observed in the previous panel, against an IMPC *Foxo1* heterozygous knock-out model, and a joint model consisting of IMPC and manual phenotypes. Ranking of diseases is by scores of the joint model against the general disease profiles.

When investigating a candidate disease-gene link, common research strategies include considering data subsets (stratification) and comparing an initial finding with additional information (augmentation). As an example of data stratification, we separated IMPC phenotypes measured in female and male animals into sex-specific models. Consistently with previous reports [26], many IMPC alleles had sex-dependent positive phenotypes. Once negative data were included, the nominal sexual dimorphism was even more pronounced (Fig 5A). This is not surprising as some phenotypes are inherently sex-dependent. Dimorphism in the raw data translated into adjusted disease scores for male and female models. On curated disease-gene pairs, scoring using only positive data did not reveal a substantial sex bias (Fig 5B). With negative data, male models yielded high scores more often than female models (Fig 5B). As an example, for Kallmann syndrome (ORPHA:478), an endocrine disorder caused by mutations in gene *Kiss1r* that affects both males and females but is diagnosed more frequently in human males, we observed that male models more closely matched the disease profile (Fig 5C). However, across all diseases, male and female models outperformed the other in certain situations and follow-up analyses should focus on individual cases. Overall, these results reveal nuances in how knock-out mice phenocopy human pathology and may help to pick suitable animals for in-depth studies.

Opposite to stratification is data augmentation, whereby an existing association is updated with new evidence. In this direction, we identified 253 alleles for which the MGI dataset provided phenotypes in addition to those screened by the IMPC. Integrating data from both sources is a means to benefit from complementary phenotyping efforts. In some cases, the MGI alleles were characterized in cross-bred mouse strains. Differences in genetic background can influence phenotypic effects of gene knock-outs [27] and integration can be a means to reduce variability. We pooled phenotypes from IMPC and MGI and repeated scoring. One example is Monosomy 9Q22.3 (ORPHA:77301), a genetic microdeletion giving rise to a developmental skeletal phenotype, which is associated to gene *Ptch1*. Even though data for this gene from individual sources resulted only in moderate scores, pooling several heterozygous models based on the IMPC allele resulted in a strong disease-gene match (Fig 5D). Thus, integrating data across laboratories can build more holistic phenotypic profiles and increase confidence in the relevance of those models.

Indeed, MGI annotations for some models are amalgamations of observations reported in more than one original publication. The incremental integration framework provides a means to track how these associations would have appeared to evolve as those original works were published. An example is a homozygous knockout in caveolin 1, *Cav1*, which is linked by orthology to Berardinelli-Seip congenital lipodystrophy (ORPHA:528), a rare condition affecting the endocrine system. Score updates for this model revealed that a high association was only achieved by pooling data from several sources (Fig 5E). Other gene-disease pairings showed similar patterns, although some scores did not saturate even after pooling several sources (S17 Fig).

Finally, we investigated how incremental integration can aid targeted research generating low-throughput data. For a case study, we selected an original article that showed that knock-down of *Foxo1*, a transcription factor, in the neutrophil population of pregnant mice can impact placental and embryo development [28]. We encoded phenotypes described in the published model into MP terms (Fig 5F) and scored these phenotypes against all diseases. We also scored an IMPC *Foxo1* knock-out model and a joint model consisting of both IMPC and manual annotations (Fig 5G, S18 Fig). The strongest match of the joint model was with Placental insufficiency (ORPHA:439167), confirming the findings in the original article. Importantly, the calculation suggested other diseases consistent with the observed phenotypes, thereby providing novel hypotheses for the functions and impact of this gene.

## Discussion

Data integration is a crucial step in piecing together knowledge of gene function, and animal models play an important role in this process. In this work, we developed an approach to scoring genotype-disease associations that incrementally incorporates observations of phenotypes into an existing score and thereby enables the tracking of associations throughout a long-term research project. The approach uses probability updates and, similarly to Bayesian models [4,29], can accommodate several types of data: repeat experiments for the same phenotype, several phenotypes for the same animal model, or different genotypes for the same intervention. In contrast to other approaches, our algorithm avoids normalization steps. This guarantees that adding data consistent with a preliminary association increases the score for that association and, similarly, that recording conflicting data weakens it. The design of our method allows for efficient [30] tracking of disease-gene associations.

As part of the initial evaluation of our approach, we performed control calculations based on curated annotations for human genetic diseases. We introduced a classification of annotation quality to highlight well-characterized diseases (diagnostic, informative classes) and reveal areas of weakness (suggestive, unspecific, unscored diseases). The boundaries between these categories are to some extent arbitrary. Nonetheless, this approach may help target curation efforts and complement ongoing evaluations of disease annotations [31]. Moreover, comparisons of human and mouse phenotypes provide insight into the ability of using animals to model particular human diseases. Given that phenotypic data help in diagnosing rare genetic diseases [32], understanding the quality of their annotation is a critical step toward increasing diagnostic rates.

With regard to mouse models, we confirmed reports that mutant mice described by the IMPC and MGI can phenocopy human diseases [13]. Our scores correlate with previously defined metrics so they can be used in the same downstream applications, for example in personalized genomic screening [32]. However, incremental scores have distinctive features. although many links between models and diseases are suggestive, the extent of their similarity is often objectively weak. We provided examples of how associations can be improved by incorporating negative results and by pooling data together from multiple sources, including bespoke experiments. The approach, therefore, pinpoints areas where most progress might be made to refine existing associations and provides a mechanism to quantify the impact of new data.

Incremental integration also has other idiosyncrasies. After integrating many supporting data, association scores can become saturated. This effect is manifest in some disease-gene pairings involving models with dozens of recorded phenotypes. Saturation conveys that an association has sound evidence and that collecting more data of the same type might have little impact. This is both a breakdown and an invitation to adopt more refined research strategies. In our efforts to classify diseases, for example, we noticed that some technical controls were concordant with many diseases. This prompted us to define profiles consisting of disease-specific phenotypes and thus describe phenotypic matching from a novel perspective. In turn, this led to a new classification of disease annotation quality and improved rankings of disease-model associations.

A possible limitation of this work in the domain of disease-matching lies in dealing with directional phenotypes [33]. We implemented fuzzy matching between phenotypes without special treatment for pairs that differ by direction. As a result, in the case study with obesity, top-ranking genes caused increases as well as decreases in body weight. Such cases provide opportunities to reason about pathways and underlying mechanisms, but it is conceivable to incorporate directionality in the scoring process.

Although we studied gene-disease associations and the discussion was grounded on animal models, the proposed framework is generalizable and extendible. It is suitable for other settings of reverse genetics [34] that collect data via several assays. It is also applicable to problems wherein data units can be encoded as terms from an ontology. Given the increasing use of log-based systems to track annotations and scientific contributions [35,36], there is scope to apply the principle of incremental data integration in other domains.

## Materials & methods

### Probability updates

The incremental integration framework proposed in this work is founded on probability updates motivated by Bayes’ formula. This transformation is most often written in terms of conditional probabilities as

$$P(f|x)=\frac{P(x|f)}{P(x)}P(f),$$

where *f* is a feature and *x* is an item of evidence supporting the feature. The probability *P*(*f*) is a prior, and *P*(*f*|*x*) is the updated value after having observed the feature in an experiment. Expanding the denominator gives

$$P(f|x)=\frac{P(x|f)}{P(x|f)P(f)+P(x|\neg f)P(\neg f)}P(f),$$

and performing some replacements of notation

P(f)=p,P(f|x)=p′,P(x|f)=τ,P(x|¬f)=ϕ,

yields

$$p\prime =\frac{\tau p}{(\tau -\phi )p+\phi }.$$

Here, *τ* is the true positive rate of the experiment and *ϕ* is the false positive rate. Thus, the update rule is parametrized by experimentally relevant quantities.

As written, the formula is applicable when *x* represents evidence that supports the feature under consideration. An update for negative evidence (an experiment that does not support the feature) follows a similar structure, except with the replacements *τ*→(1−*τ*) and *ϕ*→(1−*ϕ*).

Although the transformation is written in terms of two parameters, it can be equivalently expressed in terms of a single variable, $\omega =\frac{\tau }{\phi }$. The transformation thus has a single degree of freedom. In what follows, however, it is convenient to track *τ* and *ϕ* separately because these have a natural interpretation with respect to experimental assays.

### Gene Annotations

Mouse gene definitions were downloaded from MGI [18]. Human gene definitions were obtained from the Human Gene Naming Consortium (HGNC) [37]. Ortholog mappings between mouse and human genes were obtained through Ensembl [38].

### Ontologies

Definitions for the Human Phenotype (HP) and Mammalian Phenotype (MP) ontologies were downloaded from the OBO foundry [39]. Translation from HP disease annotations into MP was performed in two steps. First, we computed similarities between all pairs of HP-MP terms using owlsim [24] without imposing any thresholds. We then scanned the pairwise similarities, ranked them by the product of information content and semantic similarity. Thus each HP term was matched with a best MP analog. In the event of ties, multiple MP terms were preserved (S2 Table).

### Disease phenotypes

Disease annotations were obtained from ORPHANET [23]. Phenotypes associated with diseases were downloaded from the HP ontology servers [40]. Phenotype penetrance was obtained from the middle of the ranges declared in the ‘Frequency’ annotation codes. The phenotype annotations did not cover the entire ORPHANET corpus; all subsequent analyses were carried out only on the subset of annotated diseases. Similar analyses are also possible for annotations from OMIM [41].

### Mouse phenotypes

Annotations of phenotypes for mouse models were downloaded from file MGI_GenePheno.rpt provided by the Mouse Genomic Informatics (MGI) web portal [18]. The phenotypes, encoded as terms in the Mammalian Phenotype (MP) ontology, were filtered to remove entries not associated with a publication and then partitioned into mouse models by genotype, i.e. a combination of mouse strain, a single knock-out allele, and allele zygosity.

Further annotations were downloaded from the “statistical-results” SOLR core of the International Mouse Phenotyping Consortium (IMPC) data portal [25]. Phenotypes were partitioned into models by allele and zygosity. Because the IMPC data contains records of negative phenotypes, we first ran this procedure recording only positive phenotypes, and then again recording both positive and negative phenotypes. Cases where multiple data entries were found for a given phenotype were replaced by an average.

### Phenotype priors

Phenotype prior probabilities were estimated from the ensemble of MGI gene models. Starting with a vector for all phenotypes populated with a dark count (in this work, 2), each MGI gene contributed a unit count for its declared phenotypes and ontology-inferred ancestors. The vector was then normalized by the number of genes. The procedure produces estimates that capture the hierarchical nature of ontology terms, with priors for specific phenotypes lower than for broader phenotypes (S3 Table). Importantly, the procedure is invariant against small changes in the ontology structure, for example addition of intermediate or leaf nodes. However, the estimate suffers from publication bias because it is based on the genes that have been studied in the literature. It is also biased on phenotypes, with some features that have been recently added into the ontology less likely to be annotated. Nonetheless, the empirical priors summarize a heterogenous pool of mouse data that includes thousands of genes and many phenotype categories. We therefore use them to approximate the probabilities that the phenotypes occur in a background population.

### Disease representations

To define MP-based profiles for human diseases, individual HP terms from the disease annotations were mapped onto best-matching MP terms using our translation table. Phenotype penetrance was carried over with a hyperbolic-tangent scaling. The effect of this scaling was to preserve penetrance values for highly informative HP/MP terms, but reduce the weight of weak HP/MP translations.

Complete disease representations were defined by propagating phenotype penetrance up the ontology graph, interpreting joining children nodes into a parent as an ‘OR’. As an example, for two sibling terms with penetrance 0.25 and 0.5, the inferred value for the parent would be 1-(1–0.25)(1–0.5). This calculation assumes that phenotypes are independent, and while this may not be the case for some phenotype combinations, it is expected to be a reasonable approximation in most cases.

Specific disease profiles were computed by comparing each disease complete profile with all other profiles using cosine similarity. Nearest neighbors were identified and averaged using a simple mean. The specific profile was defined as the difference between the complete profile and the neighbor average, but a post-processing step was also applied. In cases when a phenotype was higher than background in the disease but the difference was lower than the background, the specific profile was set equal to the background.

### Model representations

For each mouse model, initial phenotypic profiles were set equal to the estimated priors. Given measurement data for a particular phenotype, we then updated the probability of that phenotype. For the MGI dataset, we treated each measurement with equal weight, assuming that experiments were carried out with a power of 0.8 and a false positive rate of 0.05. This assumption may not always be appropriate, but corresponds to common criteria used in experiment design. For IMPC data, we used the same settings, except for phenotypes for which we found multiple records. In those cases, the false positive rate was kept at 0.05 and the power was adjusted down from 0.8 to account for mixed evidence. This procedure is *ad-hoc*, but guarantees that the directionality of each phenotype is consistent among models based only on positive phenotypes and models based on both positive and negative data, as well as with reports on the IMPC data portal.

### Scoring models against disease profiles

Scoring of models against disease profiles was performed using the Bayesian update. Whereas earlier we used the transformation to track the probability of one phenotype, here a similar approach was used to score disease-gene associations. The prior for the association was set to an initial small value, and updates were performed taking cues from the model concise phenotypes using a three step procedure.

The first step compares a model phenotype value (*m*), a phenotype prevalence as recorded in the reference profile (*r*), and a frequency of the phenotype in a background population (*b*), which was described in a previous section. The comparison between *m* and *b* can yield three possible outcomes (greater than, less than, or equal). The same is true for *r* and *b*. Thus, each stepscan result in one of nine scenarios. Of these, true-positive (TP), false-positive (FP), true-negative (TN), and false-negative (FN) outcomes justify an update to the model-disease association score.

The second step in the calculation determines initial estimates for *τ* and *ϕ* by taking into account values from the model (*m*) and the background (*b*) (Table 1).

**Table 1:** Parameters for the Bayes update formula.

Scenario	Outcome	***τ***	***ϕ***
*m*>*b*,*r*>*b*	TP	*m*	*b*
*m*>*b*,*r*<*b*	FP	*τ*_*a*_(1−*m*)	*ϕ*_*a*_(1−*b*)
*m*<*b*,*r*<*b*	TN	1−*m*	1−*b*
*m*<*b*,*r*>*b*	FN	1−*b*	1−*m*

While most cases have straightforward definitions, handling of false-positives (FP) involves determining quantities *τ*_*a*_ and *ϕ*_*a*_−we can come back to these details later.

The third and last step of the procedure adjusts the initial estimates for *τ* and *ϕ* using penetrance recorded in the reference. This is performed through an interpolation factor,

$$\alpha =\frac{r-b}{\Theta (r-b)-b},$$

where *Θ*(*r*−*b*) is a step function that evaluates to 1 when *r*>*b* and to 0 when *r*≤*b*. This ratio is then used to redefine the *τ* estimate in the previous step,

τ→ατ+(1−α)ϕ.

The overall effect is to set *τ* = *ϕ* when the phenotype value in the reference is equal to the background, leave it unchanged when the reference is maximally different from the reference, or perform a linear interpolation.

Returning to the case of false positives (FPs), the quantities *τ* and *ϕ*_*a*_ are computed as follows. For a given seed phenotype, the algorithm determines an ancestral phenotype that has a value greater than background in the reference. Given the ancestral phenotype and its background level, *b*_*a*_, it is possible to estimate how the upregulation observed in the model would impact that ancestral phenotype,

$$m_{a}=\frac{\tilde{\omega }b_{a}}{(\tilde{\omega }-1)b_{a}+1},\;\mathrm{where}\;\tilde{\omega }=\left(\frac{b}{1-b}\right)\left(\frac{1-m}{m}\right).$$

Values *m*_*a*_ and *b*_*a*_ would be suitable for performing a score update in the style of a true-positive. However, in order to account that the match is based on an ancestral phenotype, *τ*_*a*_ and *ϕ*_*a*_ are set

$$\tau_{a}=(1-\beta )m_{a}+\beta b_{a},\;and\;\phi_{a}=b_{a},$$

where *β* is another interpolation factor. In this work, we use $\beta =tanh\;({fp}_{penalty}log\frac{b_{a}}{b})$, with *fp*_*penalty*_ a free parameter. In practice, this interpolation factor is close to unity when the ancestral phenotype differs substantially from the seed phenotype. After computing *τ*_*a*_ and *ϕ*_*a*_, the algorithm returns to the main flow and assesses a weighting due to the prevalence of the reference using the interpolation factor *α*, except using *r*_*a*_ instead of *r* in the numerator.

Overall, each of the updates is dependent only on the values associated with a phenotype or an ancestor. The calculations are decoupled from technical details of the ontology such as number of nodes and number of children. This is in contrast to schemes that use ontologies in a direct manner–for example, using Jaccard indexes between sets of ontology terms or likelihoods computed via probability products–which are necessarily responsive to changes in administrative aspects of the ontology such as addition of intermediate nodes. Invariance to such changes ensures that our algorithm emphasizes biological meaning of the model-disease comparison over ontological book-keeping.

### Calibration

To determine appropriate values for the free parameters in our method, we matched diseases to mouse models with a knock-out in a disease-causing gene. We also matched the same diseases to alternative randomly selected models with a similar phenotypic profile, i.e. the same number of phenotypes as the model with the disease-causing gene. These sets, which are positive and negative controls, were then scored at various settings. We explored one parameter that determines how missing annotations in the references are encoded with respect to the background, and one parameter that determines the strength of a penalty during handling of FP comparisons.

### Visualization of the disease phenotype landscape

Visualization of phenotypic similarities across diseases was performed using a two-pass protocol. In a first pass, we computed the sums of the disease complete representations to identify well-annotated diseases. The subset of diseases with high sums were embedded onto a 2D plane using UMAP [42,43] with a cosine metric. In a second pass, the remaining diseases were injected into the embedding. Thus, the disease landscape map was determined by well-annotated diseases, but finally includes all diseases.

### Stratification and integration

Stratification models were obtained from the IMPC dataset using the same strategies as above, but with an additional step segrating phenotypes by male and female labels.

Integration models were defined and scored separately. Joint models were defined in cases when a single allele-zygosity pair was detected in both the IMPC and MGI datasets. Model phenotypes for the joint models were defined by concatenating the two datasets. During the stage converting from raw data into a concise model representation, phenotype probabilities were updated as many times as there were measurements, thus giving confirmed phenotypes larger weight than single-measured features.

To assess the evolution of phenotype annotations in the MGI dataset, we associated each phenotype with a Pubmed ID. Most phenotype-publication pairing in the raw data were straightforward, but some cases were ambiguous with multiple pubmed ids listed together. For this analysis, the publication with the lowest pubmed id was selected. Because of the imperfection of this procedure and its potential to miss contributions from certain publications, the publication identifiers were all hidden in the figures.

### Software

The software for preparing raw data and for computing disease-model association scores is available at www.github.com/tkonopka/phenoscoring. Follow-up analyses were performed using custom bash and R scripts [44].

### Ethics

This work relies on open-access and public data. Ethics of animal experiments referred to in the text are described in the original data sources.

## Supporting information

S1 Fig Evolution of integrative scores. (A) A hypothetical scenario in which two experimental outcomes are available at an initial time (first row), and further evidence becomes available at later times (second and third row). Summary metrics are displayed on the right hand side. All measurements are in arbitrary units. (B) Evolution of summary statistics in the scenario from (A) as a function of time, showing undesirable decrease in summary scores as positive data is integrated. (C) Evolution of an alternative score showing a desirable increase as positive data is integrated. One line is computed using the order of data presentation from (A); a second line shows evolution based on a permutation.
(TIFF)

S2 Fig Properties of update-based scoring. (A) Evolution of a score as supportive (+) and contradictory (-) data become available. Lines illustrate a few permutations of the order in which the evidence is presented; in all cases, contradictory data lead to score decreases, but scores can nonetheless remain high if the the balance between supportive and contradictory evidence is positive. (B) Evolution of a score as supportive evidence becomes available. Lines illustrate evolutions when the data is strong or weak; in both cases, scores eventually approach the maximal possible value, 1.
(TIFF)

S3 Fig Cross ontology mapping. Correlations between sums over disease representations expressed in the original human phenotype (HP) ontology and the translation into the mammalian phenotype (MP) ontology. Correlations are computed using (A) complete and (B) specific representations.
(TIFF)

S4 Fig Disease annotations. Correlations between the sum over complete disease representations and a sum over the information content of disease concise phenotypes. Information content is defined as minus of the log of the phenotype prevalence.
(TIFF)

S5 Fig Disease annotations by disease area. (A) Disease areas, listed in order of number of diseases belonging to that area (K, thousands). Some diseases can be annotated to more than one area. Additional areas exist in the annotation set, but have fewer diseases. (B) Distribution of sums over general phenotype representations of diseases, split by number of disease areas. (C) Analogous to previous panel, with sums over specific phenotype representations. Numbers in right-hand margins indicate the number of diseases in each stratum.
(TIFF)

S6 Fig Disease Landscape. All panels display diseases arranged on a plane according to their phenotypic similarity. Each panel highlights diseases matching a disease category (a disease can belong to more than one category).
(TIFF)

S7 Fig Classification of diseases by annotation quality. Technical controls are synthetic models constructed from disease annotations. Each control is derived from one disease and contains one measurement for each phenotype associated with the disease. (A) Scores of technical controls against their matched disease, performed using the MP ontology. (B-D) Examples of control models scored against one disease (labeled at the top), ranked using the general score. In (B), the matched control (dark bar) is the best scoring model and other models (light bars) all score lower. In (C) and (D), the best scoring models originate from other diseases and the matched model is not present in the top hits. The annotations for those two diseases are thus not diagnostic. (E) Summary of disease scores according to the scores with their matched technical control. Diagnostic diseases are informative diseases for which the matched control is the best scoring technical control. (F-J) Analogous visualization to (A-E), except that all calculations were performed in the space of the Human Phenotype (HP) ontology, without translation.
(TIFF)

S8 Fig Effect of FP-penalty on disease classification. Summaries of disease classification based on MP ontology evaluated using different penalties for scoring false-positive comparisons.
(TIFF)

S9 Fig Effect of nearest neighbors on disease classification. Summaries of disease classification evaluated using different numbers of nearest neighbors.
(TIFF)

S10 Fig Phenotype measurements in MGI and IMPC datasets. (A) Distribution (histogram) of the number of measurements per mouse model in the MGI dataset; some genes may be associated with several models and thus can be overrepresented in the histogram. (B) Distribution of number of positive phenotypes in the IMPC dataset. (C) Distribution of negative phenotypes in the IMPC dataset. (D) Summary of positive and negative phenotypes in IMPC mouse models.
(TIFF)

S11 Fig Calibration of free parameters with MGI data. Known (curated) disease-gene pairs were scored at various settings to assess the impact of two free parameters: the reference background multiplier (bg) and the false positive penalty (fp). As comparison, a corresponding (control) set of disease-gene pairs were selected at random using models with equivalent numbers of phenotypes. All calculations were performed using MGI models against disease general (G) and specific (S) profiles. Boxes plots display the median enrichment (center line), interquartile range (rectangles) and the 0.05 and 0.95 quantiles (whiskers).
(TIFF)

S12 Fig Calibration of free parameters with IMPC data. Similar to previous figure, here using IMPC models with (A) only positive phenotypes and (B) with both positive and negative phenotypes.
(TIFF)

S13 Fig Comparison with phenodigm scores. Points denote a disease-gene pairs recorded in disease annotations. Phenodigm scores, computed accordingly to a published algorithm, are compared with incremental scores using the disease (A) general and (B) specific profiles. Colors indicate the number of phenotypes in the disease complete representation. All scores are computed using MGI phenotypes.
(TIFF)

S14 Fig Trends in disease-gene association scores. Known disease-gene associations are scored using mouse model data from the MGI database. (A) Phenodigm scores, computed using a published algorithm, are negatively correlated with the extent of disease annotation. (B) Incremental scores of models against general disease profiles have a positive correlation with the extent of disease annotation. (C) Phenodigm scores have a positive correlation with the extent of model annotation, but regress to an average score for well-annotated models. (D) Incremental scores against general disease profiles increase with the extent of model annotation. All correlations are computed with the spearman method. Lines represent spline fits.
(TIFF)

S15 Fig Correlations with annotation extent. All panels show correlations of sums of model scores against disease profiles. Sums of scores provide an estimate for the number of genes that are phenotypically similar to a disease. Horizontal axes shows the extent of disease annotation, measured by a sum of phenotype values in disease complete representations. Panels show comparisons based on (A) IMPC models scored against the general disease profiles, (B) IMPC models against specific disease profiles, (C) MGI models scored against general disease profiles, and (D) MGI models scored against specific disease profiles. Sub-panels show the same data—one with a logarithmic vertical axis and one with a standard axis.
(TIFF)

S16 Fig Disease-gene associations from MGI models. (A) Visualization of disease with many well-scored genes on the disease landscape map. The spread of highlighted dots on the map indicates that the disease set contains disease pertaining to a wide range of human pathology. (B) Statistical comparison of the multigenic disease set with curated disease categories. Heatmap boxes show overlap of the disease set with disease categories. Statistically unusual comparisons, evaluated using a Fisher test, are highlighted.
(TIFF)

S17 Fig Scoring of MGI models informed by more than one publication. (A) Correlation between the number of publications associated with a model and the number of its distinct phenotypes. (B) Incremental scoring for models with the most publications and involving a known gene-disease associations. The horizontal axis tracks phenotypes, grouped by source publications (notches). Lines reveal scores against the general and specific profiles. Disease annotation quality is indicated in the titles. Interestingly, one panel shows that limited information about disease phenotypes limits mouse models to achieve high scores.
(TIFF)

S18 Fig Scoring using manually curated phenotypes. (A) Comparison of general scores computed for an IMPC model with Foxo1 knockout and a joint model consisting of IMPC and manually annotated phenotypes. (B) Top-ranking diseases for three related models involving gene Foxo1: phenotypes observed in an IMPC knock-out mouse (IMPC), manually curated phenotypes (MANUAL), and a joint model combining the IMPC and manual data (JOINT). (C) Analogous to (A), showing scores against specific disease profiles. (D) Analogous to (B), showing rankings against specific disease profiles.
(TIFF)

S1 Table Disease summary. Assessment of ORPHANET disease annotations.
(TSV)

S2 Table Cross ontology mapping. Translation from HP to MP ontology terms computed by owlsim, v0.3.0. The score column indicates the produce of term overlap and information content for a match. The table displays a single best-matching MP term per HP term; multiple terms are given only in the case of equal scores among the best matches.
(TSV)

S3 Table Phenotype priors. Prior probabilities for MP ontology terms estimated from MGI annotations.
(TSV)
