# Supplementary figures and images for: Incremental data integration for tracking genotype-disease associations
Source: PLoS Comput Biol. 2020 Jan 27;16(1):e1007586. doi: 10.1371/journal.pcbi.1007586 (PMC7004389; doi:10.1371/journal.pcbi.1007586)

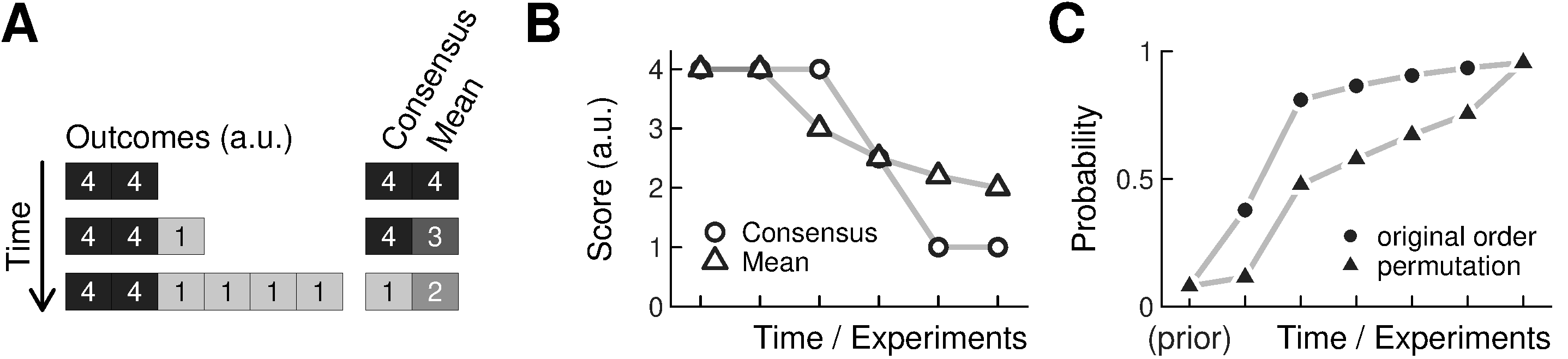

Supplement: S1 Fig — (A) A hypothetical scenario in which two experimental outcomes are available at an initial time (first row), and further evidence becomes available at later times (second and third row). Summary metrics are displayed on the right hand side. All measurements are in arbitrary units. (B) Evolution of summary statistics in the scenario from (A) as a function of time, showing undesirable decrease in summary scores as positive data is integrated. (C) Evolution of an alternative score showing a desirable increase as positive data is integrated. One line is computed using the order of data presentation from (A); a second line shows evolution based on a permutation. (TIFF) [file pcbi.1007586.s001.tiff]

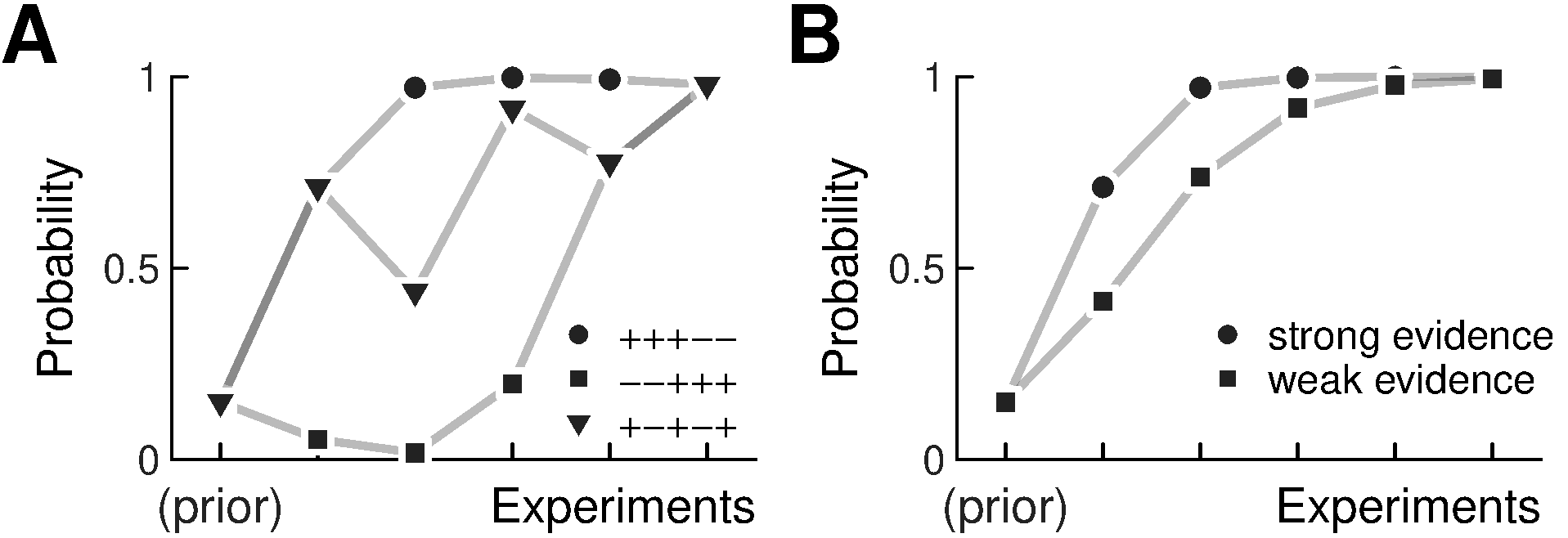

Supplement: S2 Fig — (A) Evolution of a score as supportive (+) and contradictory (-) data become available. Lines illustrate a few permutations of the order in which the evidence is presented; in all cases, contradictory data lead to score decreases, but scores can nonetheless remain high if the the balance between supportive and contradictory evidence is positive. (B) Evolution of a score as supportive evidence becomes available. Lines illustrate evolutions when the data is strong or weak; in both cases, scores eventually approach the maximal possible value, 1. (TIFF) [file pcbi.1007586.s002.tiff]

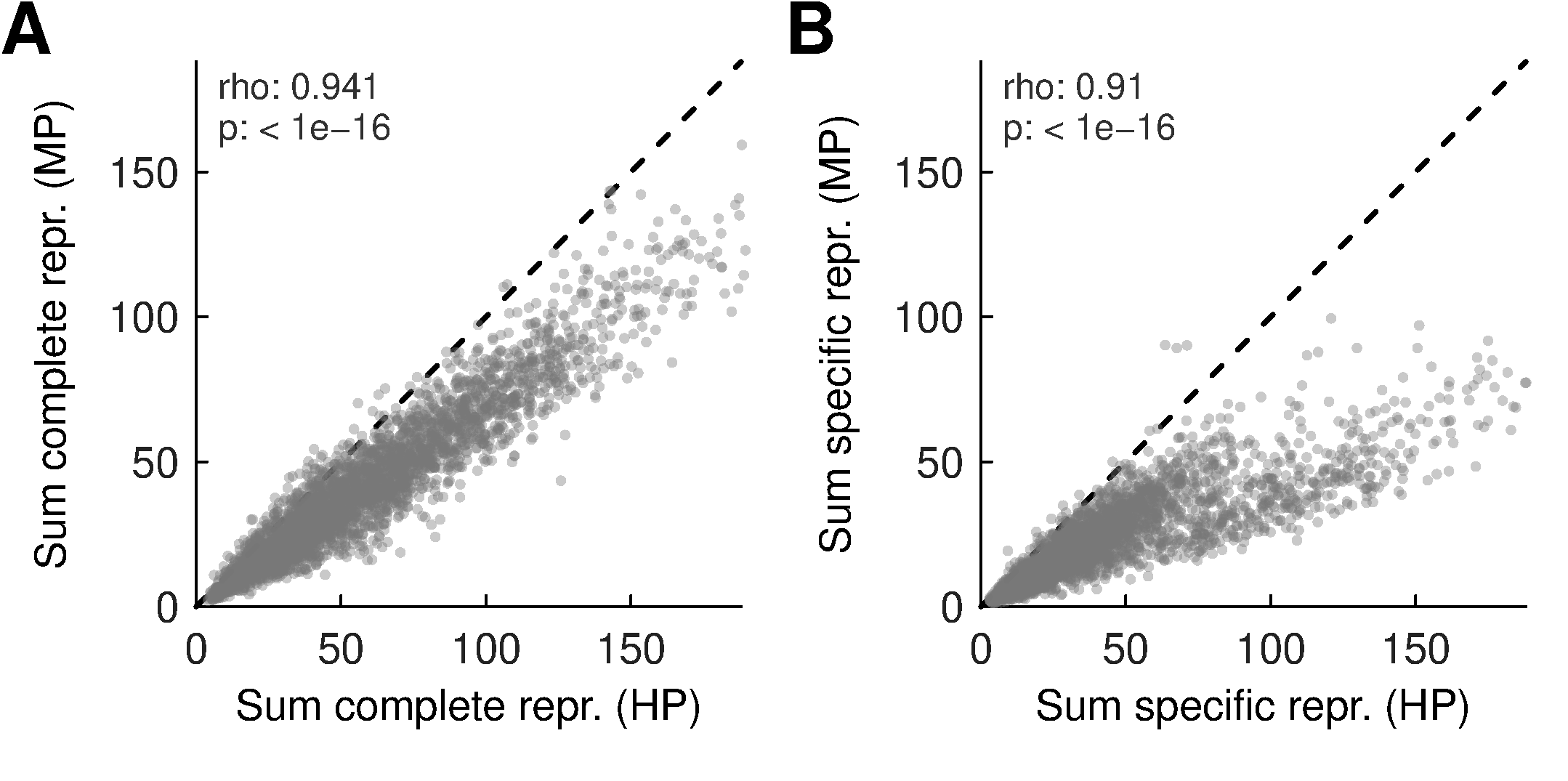

Supplement: S3 Fig — Correlations between sums over disease representations expressed in the original human phenotype (HP) ontology and the translation into the mammalian phenotype (MP) ontology. Correlations are computed using (A) complete and (B) specific representations. (TIFF) [file pcbi.1007586.s003.tiff]

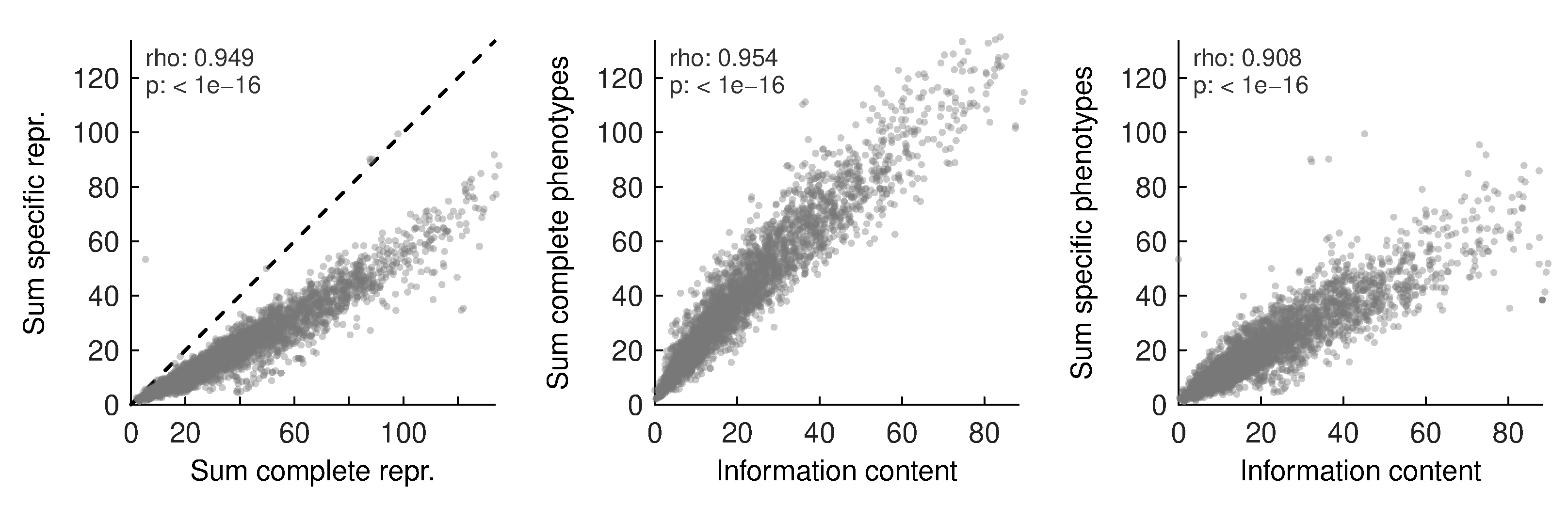

Supplement: S4 Fig — Correlations between the sum over complete disease representations and a sum over the information content of disease concise phenotypes. Information content is defined as minus of the log of the phenotype prevalence. (TIFF) [file pcbi.1007586.s004.tiff]

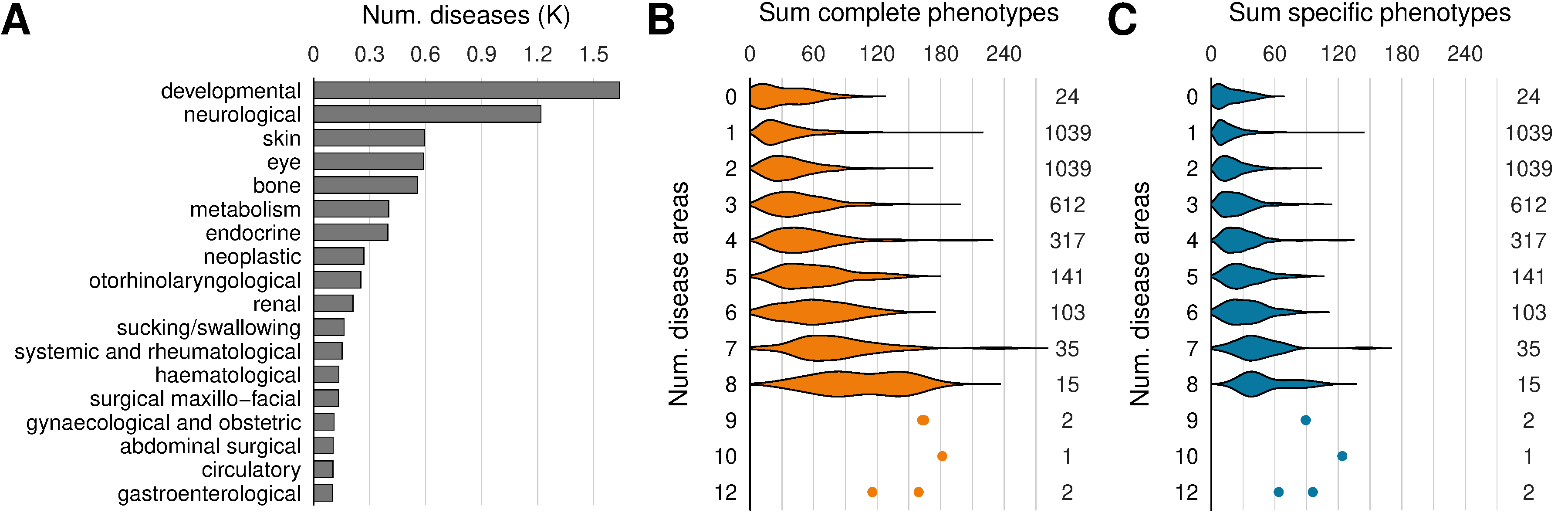

Supplement: S5 Fig — (A) Disease areas, listed in order of number of diseases belonging to that area (K, thousands). Some diseases can be annotated to more than one area. Additional areas exist in the annotation set, but have fewer diseases. (B) Distribution of sums over general phenotype representations of diseases, split by number of disease areas. (C) Analogous to previous panel, with sums over specific phenotype representations. Numbers in right-hand margins indicate the number of diseases in each stratum. (TIFF) [file pcbi.1007586.s005.tiff]

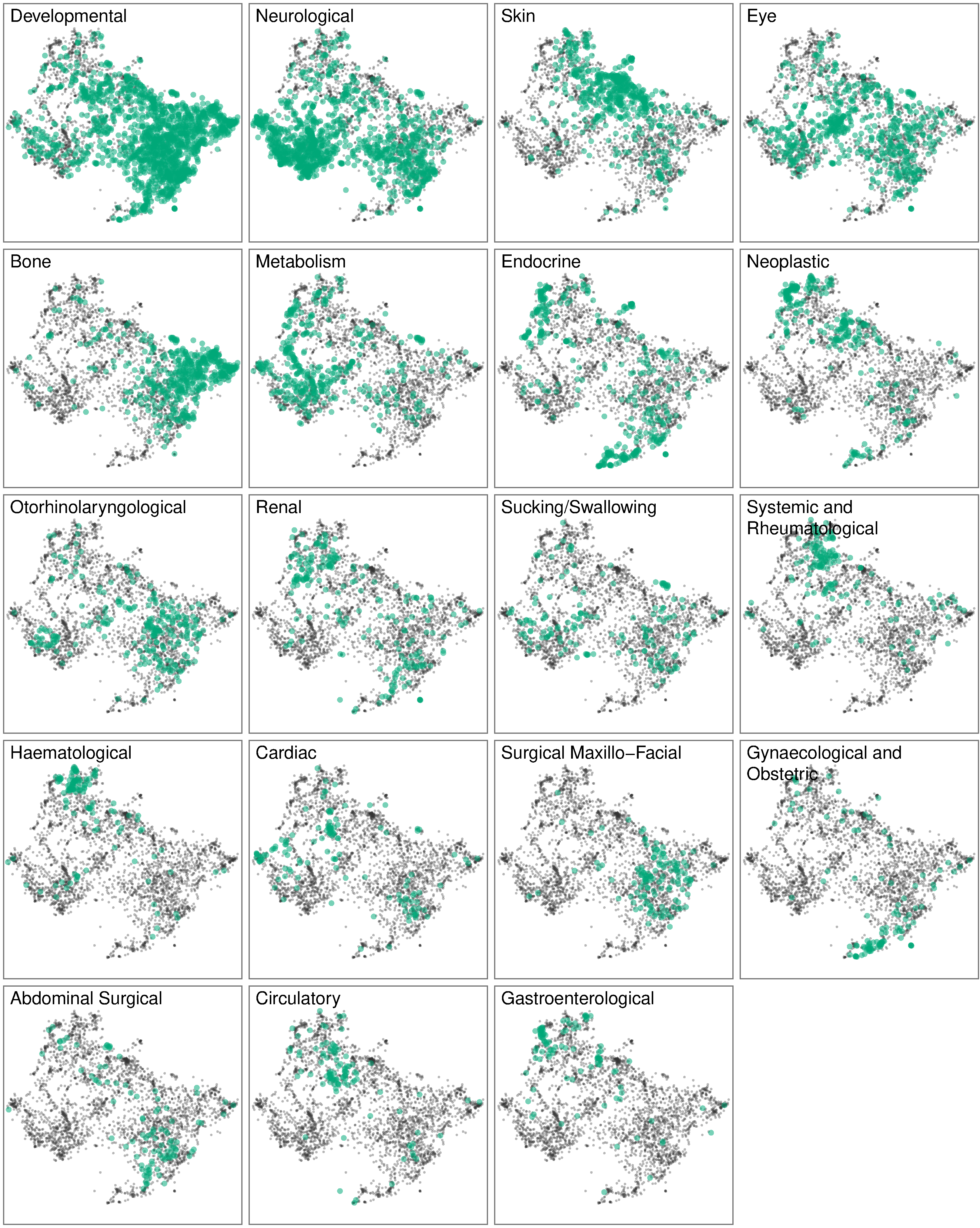

Supplement: S6 Fig — All panels display diseases arranged on a plane according to their phenotypic similarity. Each panel highlights diseases matching a disease category (a disease can belong to more than one category). (TIFF) [file pcbi.1007586.s006.tiff]

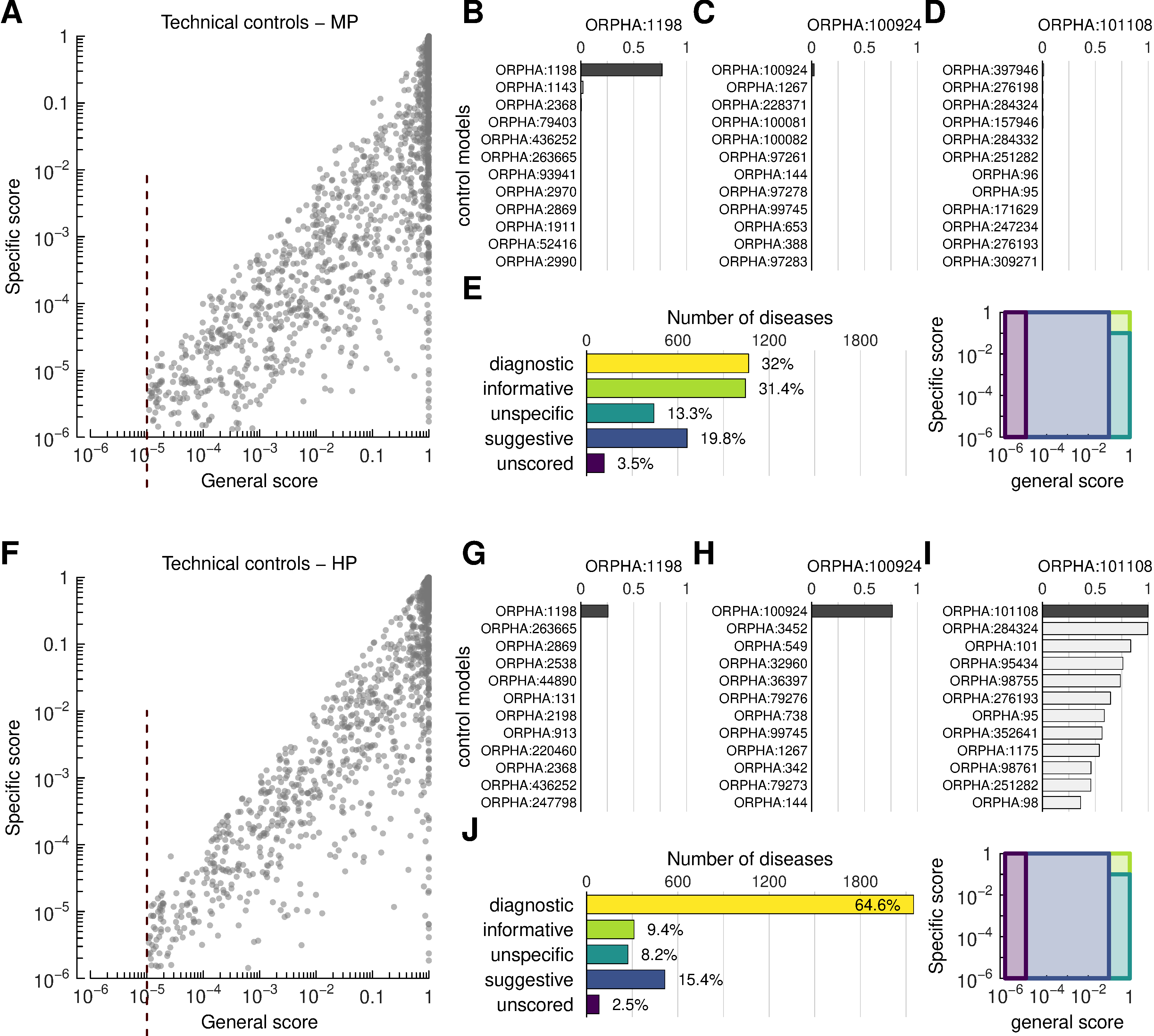

Supplement: S7 Fig — Technical controls are synthetic models constructed from disease annotations. Each control is derived from one disease and contains one measurement for each phenotype associated with the disease. (A) Scores of technical controls against their matched disease, performed using the MP ontology. (B-D) Examples of control models scored against one disease (labeled at the top), ranked using the general score. In (B), the matched control (dark bar) is the best scoring model and other models (light bars) all score lower. In (C) and (D), the best scoring models originate from other diseases and the matched model is not present in the top hits. The annotations for those two diseases are thus not diagnostic. (E) Summary of disease scores according to the scores with their matched technical control. Diagnostic diseases are informative diseases for which the matched control is the best scoring technical control. (F-J) Analogous visualization to (A-E), except that all calculations were performed in the space of the Human Phenotype (HP) ontology, without translation. (TIFF) [file pcbi.1007586.s007.tiff]

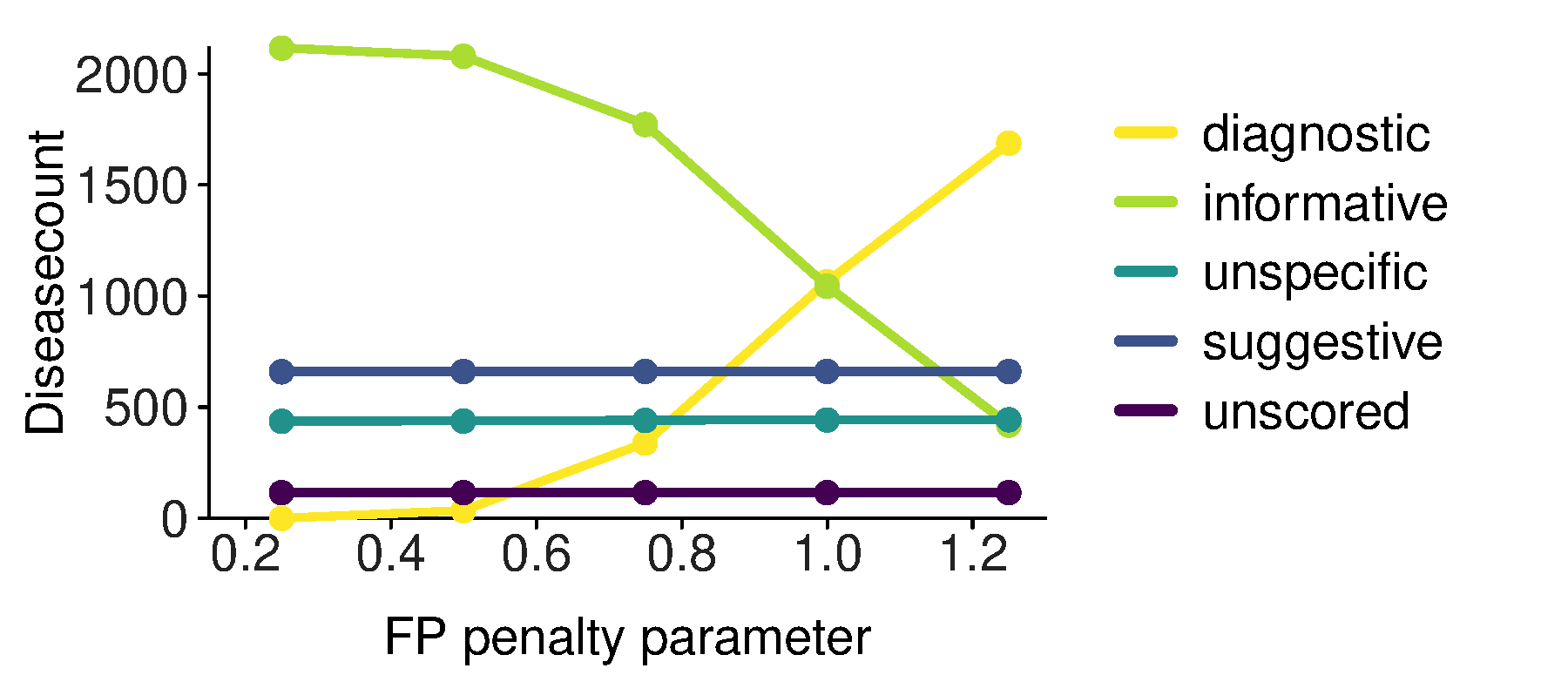

Supplement: S8 Fig — Summaries of disease classification based on MP ontology evaluated using different penalties for scoring false-positive comparisons. (TIFF) [file pcbi.1007586.s008.tiff]

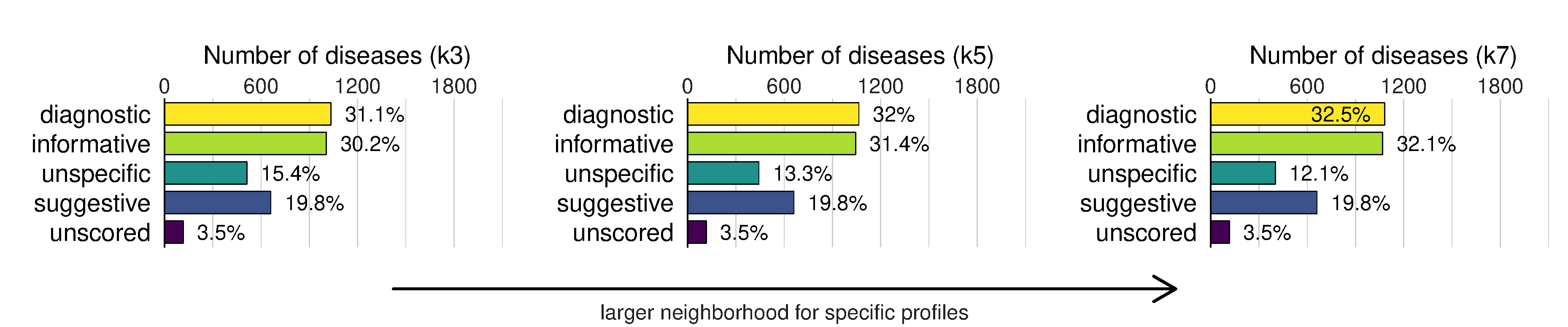

Supplement: S9 Fig — Summaries of disease classification evaluated using different numbers of nearest neighbors. (TIFF) [file pcbi.1007586.s009.tiff]

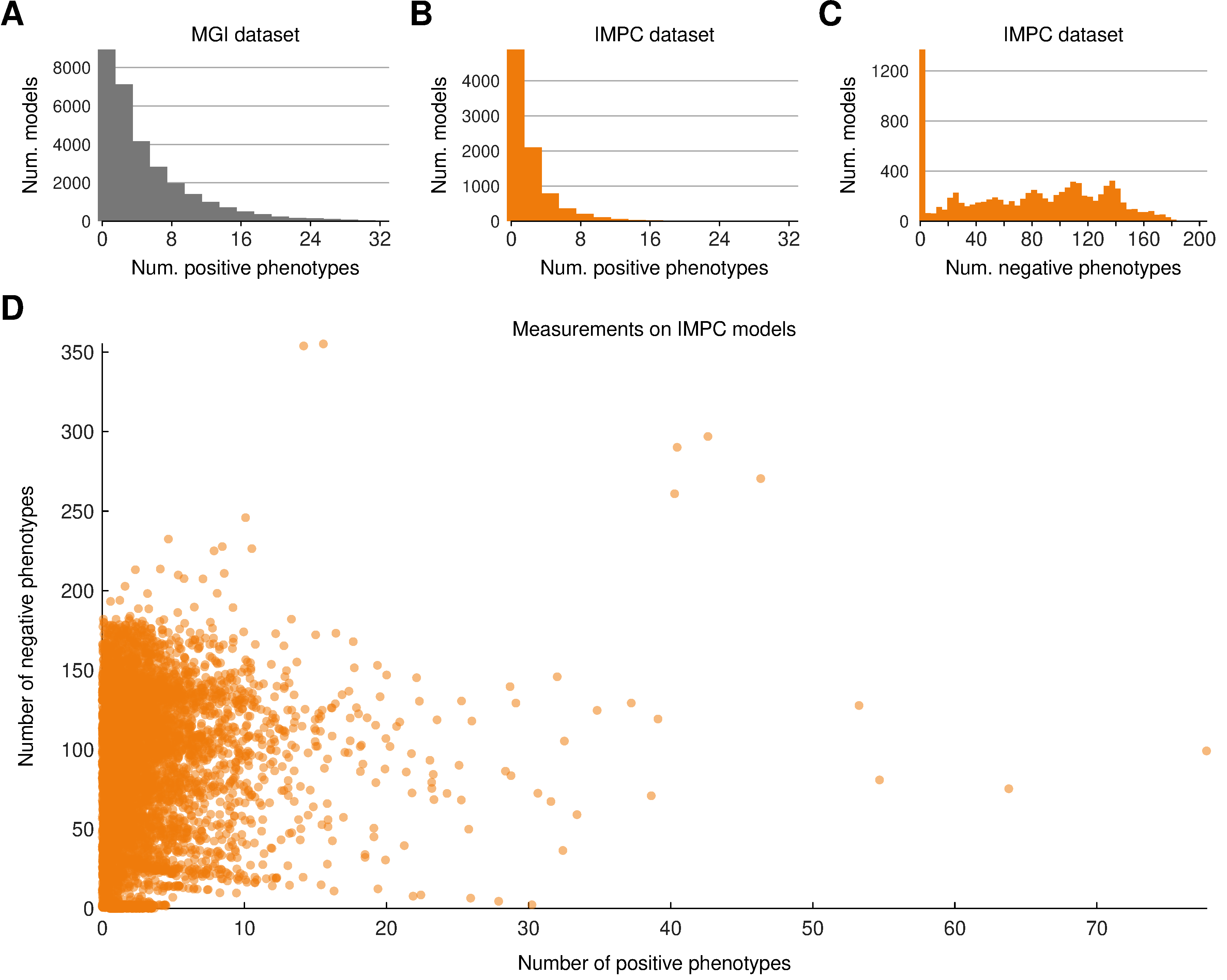

Supplement: S10 Fig — (A) Distribution (histogram) of the number of measurements per mouse model in the MGI dataset; some genes may be associated with several models and thus can be overrepresented in the histogram. (B) Distribution of number of positive phenotypes in the IMPC dataset. (C) Distribution of negative phenotypes in the IMPC dataset. (D) Summary of positive and negative phenotypes in IMPC mouse models. (TIFF) [file pcbi.1007586.s010.tiff]

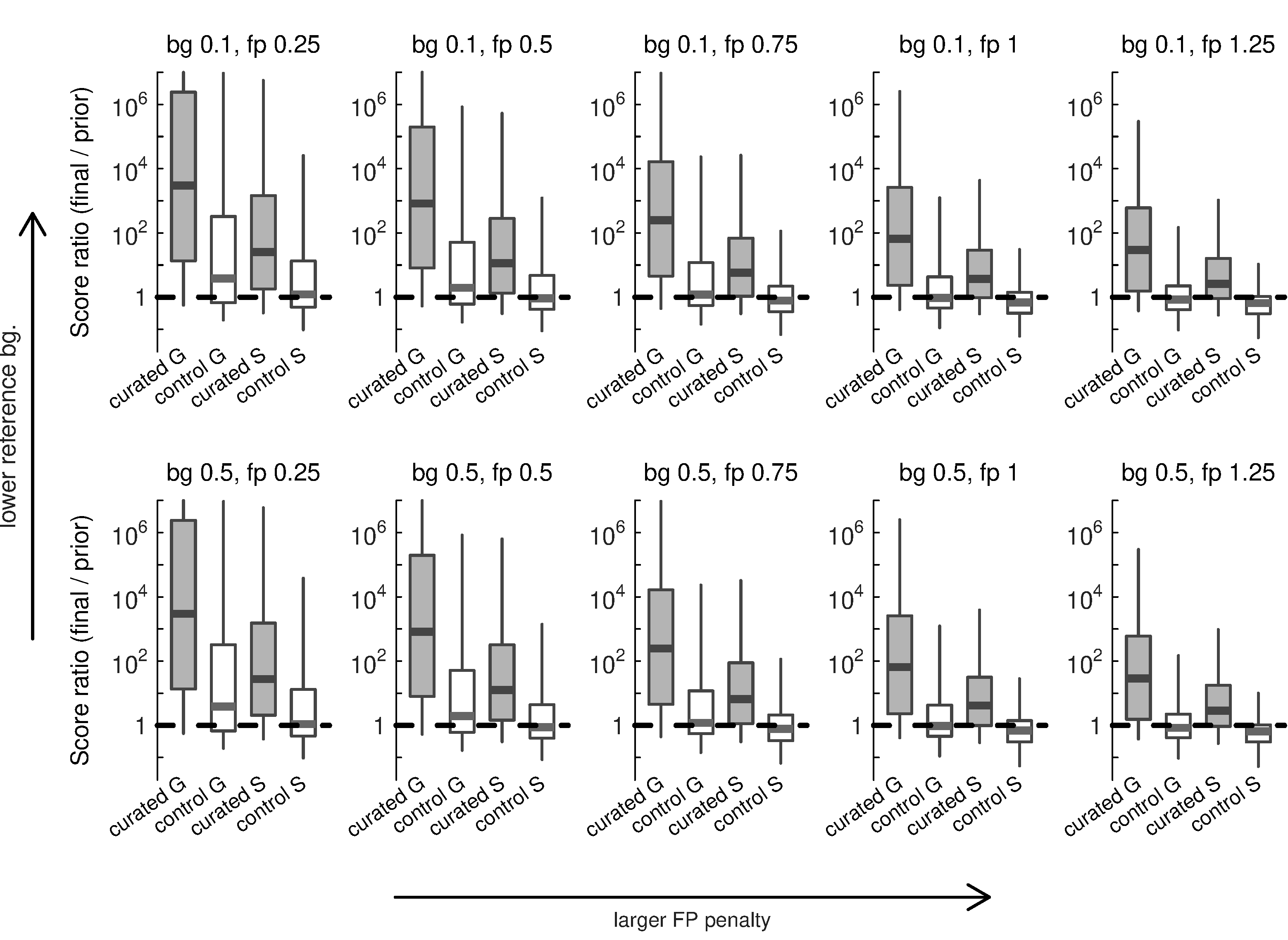

Supplement: S11 Fig — Known (curated) disease-gene pairs were scored at various settings to assess the impact of two free parameters: the reference background multiplier (bg) and the false positive penalty (fp). As comparison, a corresponding (control) set of disease-gene pairs were selected at random using models with equivalent numbers of phenotypes. All calculations were performed using MGI models against disease general (G) and specific (S) profiles. Boxes plots display the median enrichment (center line), interquartile range (rectangles) and the 0.05 and 0.95 quantiles (whiskers). (TIFF) [file pcbi.1007586.s011.tiff]

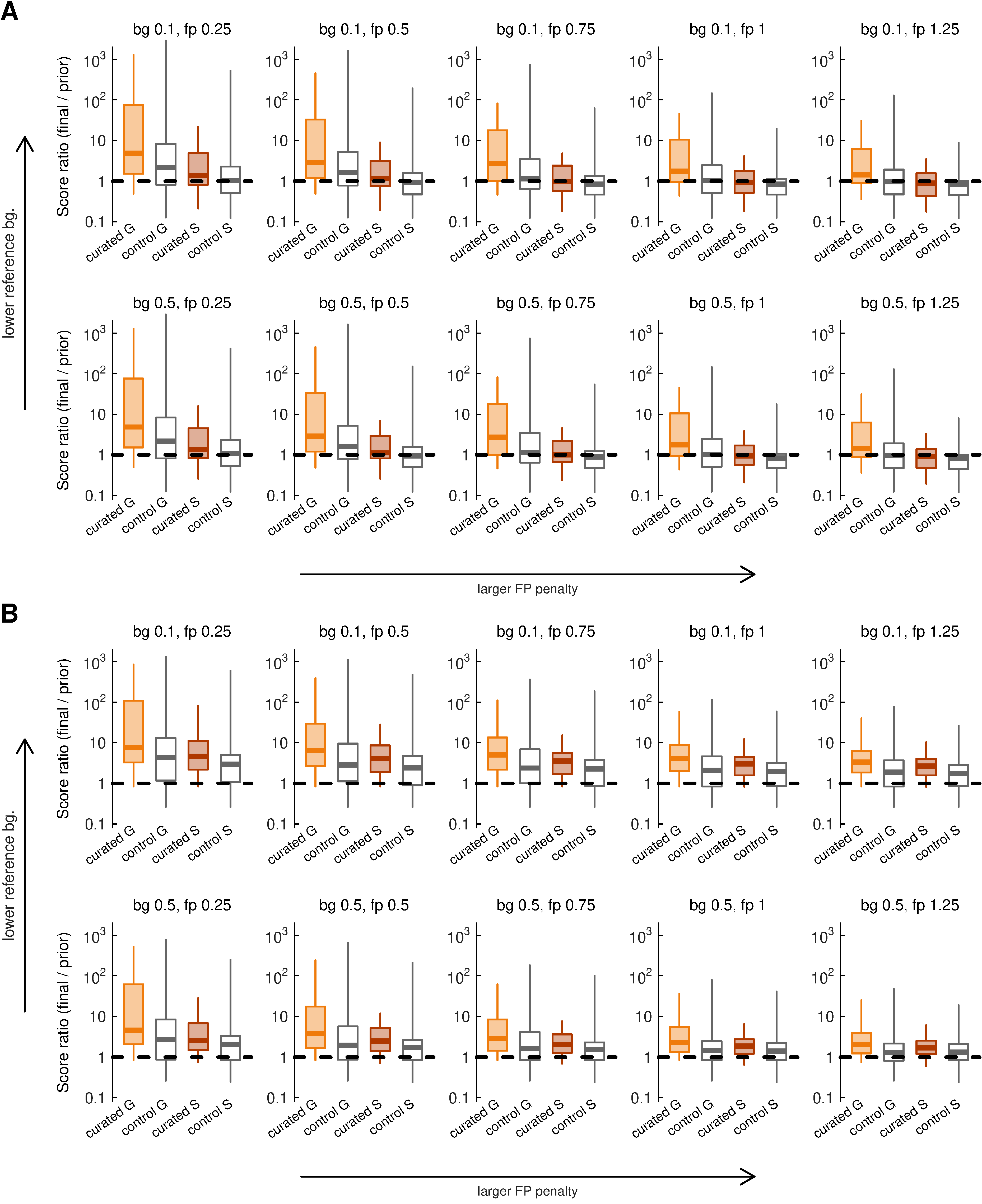

Supplement: S12 Fig — Similar to previous figure, here using IMPC models with (A) only positive phenotypes and (B) with both positive and negative phenotypes. (TIFF) [file pcbi.1007586.s012.tiff]

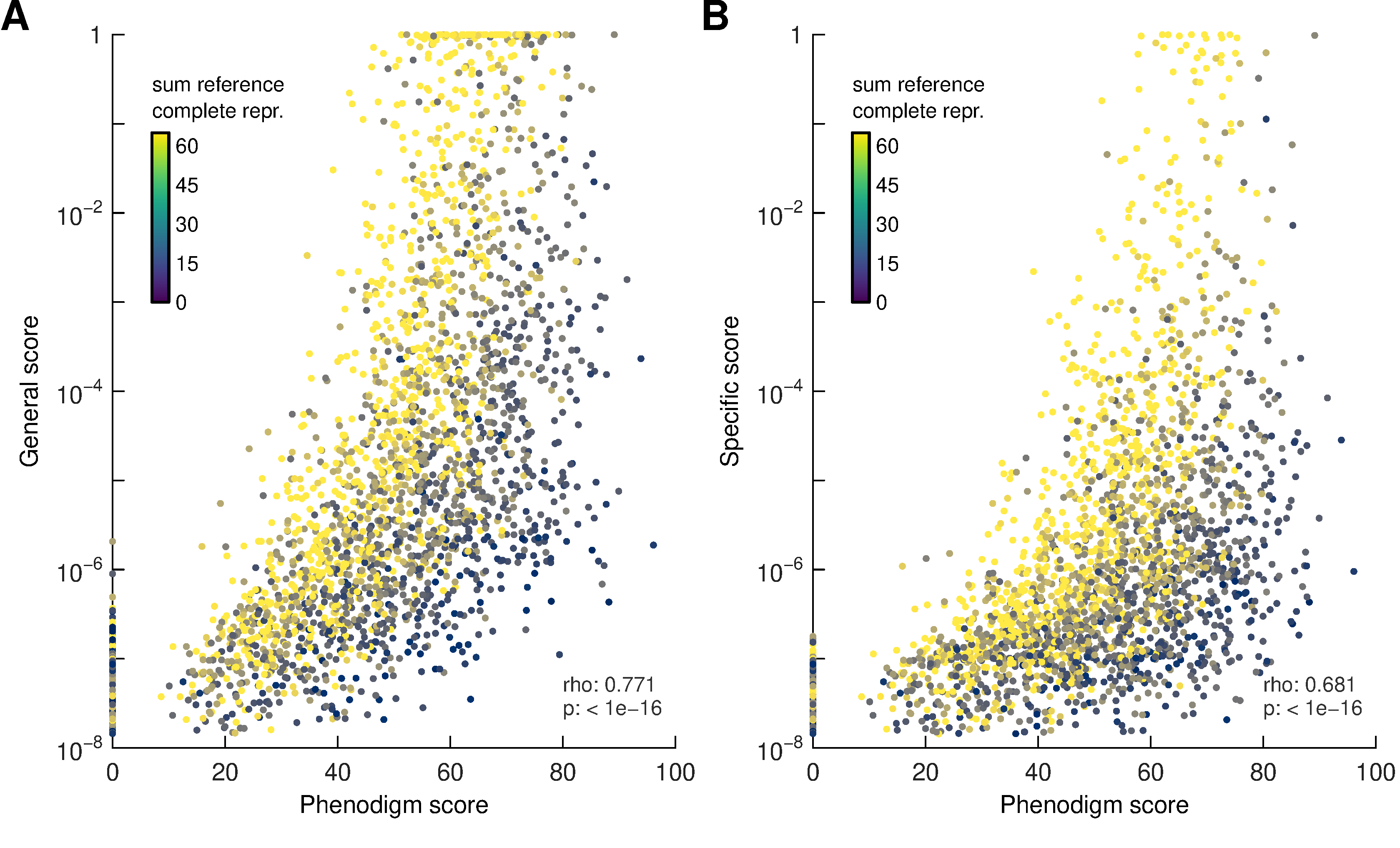

Supplement: S13 Fig — Points denote a disease-gene pairs recorded in disease annotations. Phenodigm scores, computed accordingly to a published algorithm, are compared with incremental scores using the disease (A) general and (B) specific profiles. Colors indicate the number of phenotypes in the disease complete representation. All scores are computed using MGI phenotypes. (TIFF) [file pcbi.1007586.s013.tiff]

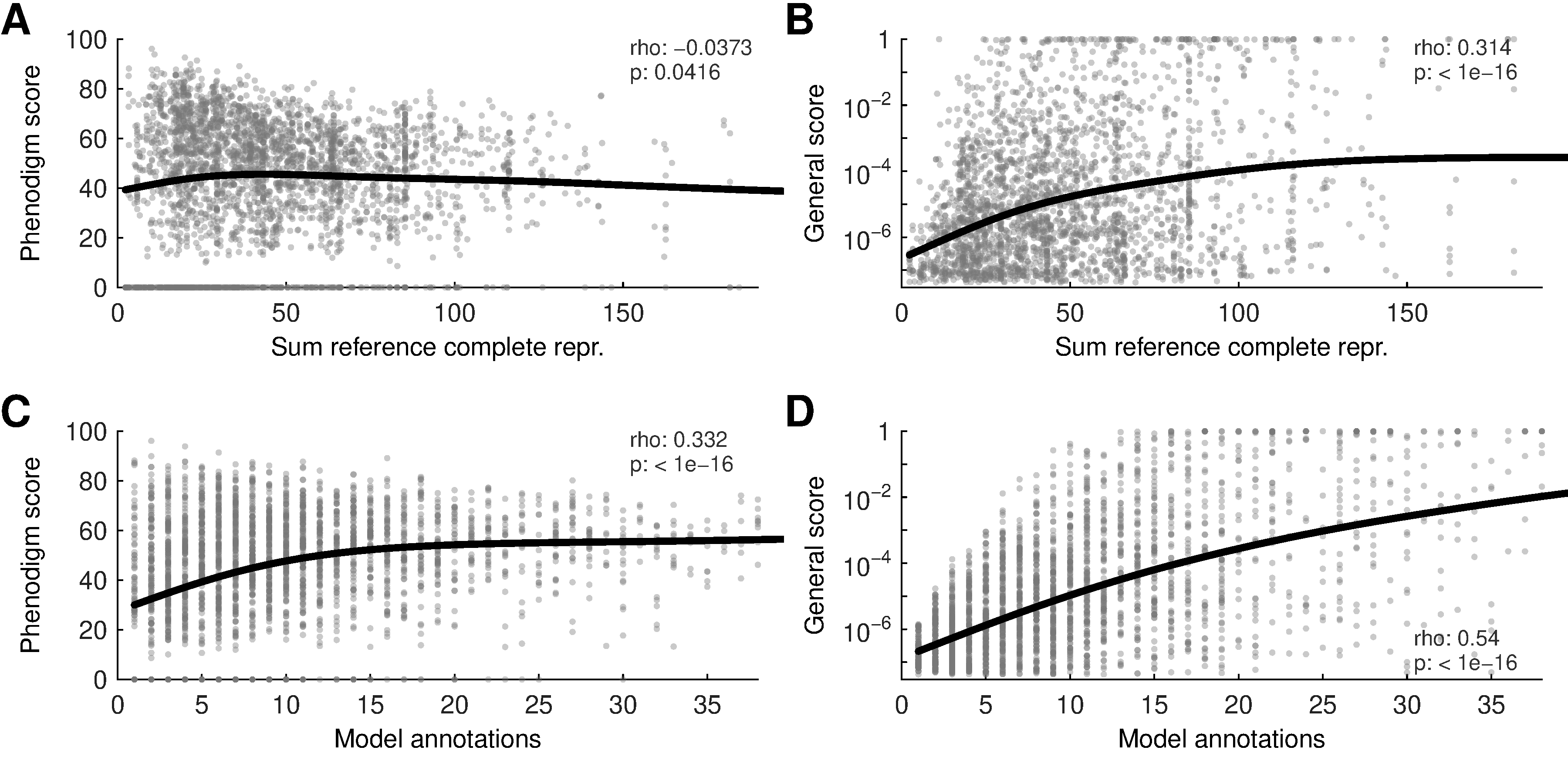

Supplement: S14 Fig — Known disease-gene associations are scored using mouse model data from the MGI database. (A) Phenodigm scores, computed using a published algorithm, are negatively correlated with the extent of disease annotation. (B) Incremental scores of models against general disease profiles have a positive correlation with the extent of disease annotation. (C) Phenodigm scores have a positive correlation with the extent of model annotation, but regress to an average score for well-annotated models. (D) Incremental scores against general disease profiles increase with the extent of model annotation. All correlations are computed with the spearman method. Lines represent spline fits. (TIFF) [file pcbi.1007586.s014.tiff]

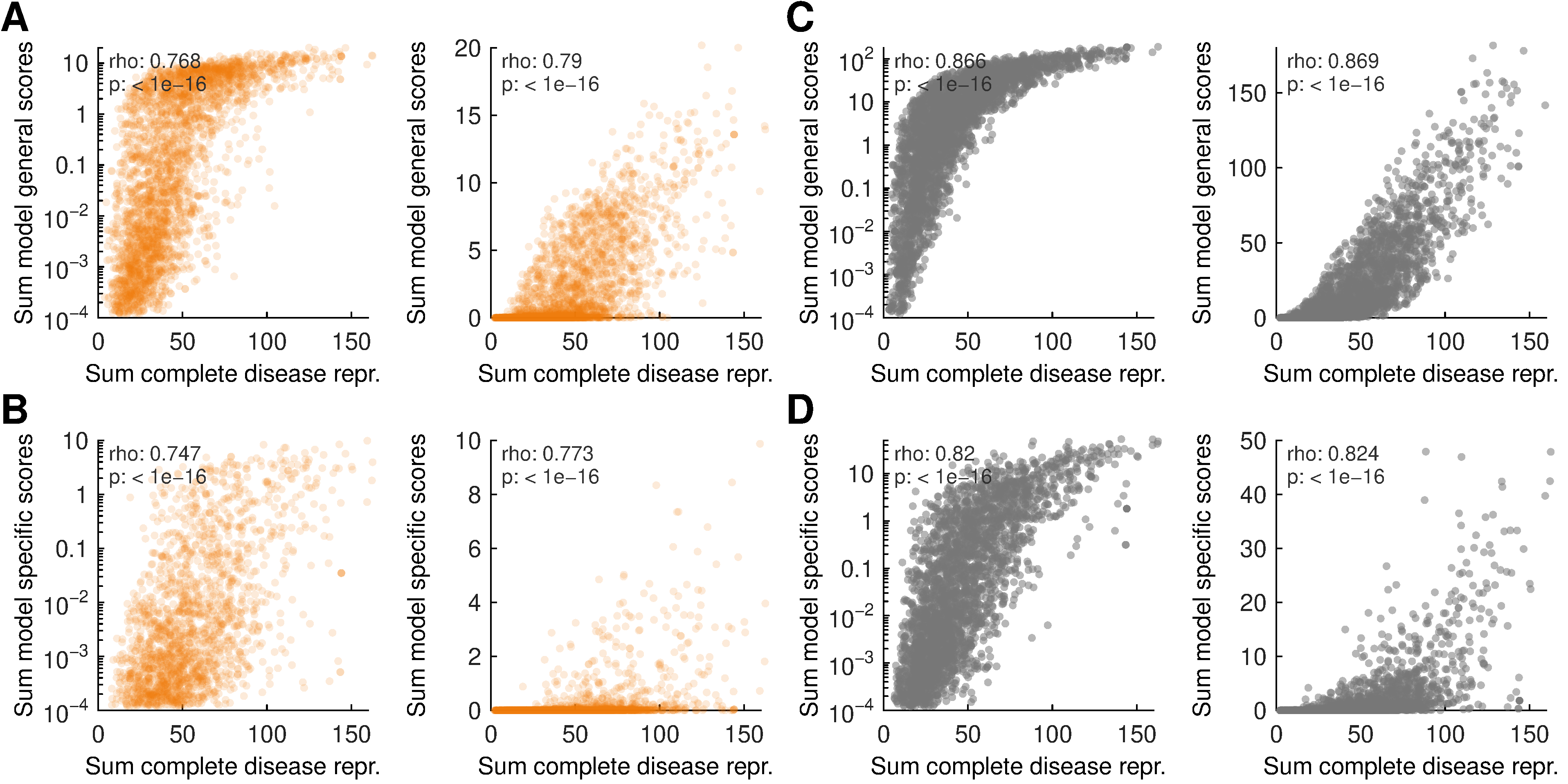

Supplement: S15 Fig — All panels show correlations of sums of model scores against disease profiles. Sums of scores provide an estimate for the number of genes that are phenotypically similar to a disease. Horizontal axes shows the extent of disease annotation, measured by a sum of phenotype values in disease complete representations. Panels show comparisons based on (A) IMPC models scored against the general disease profiles, (B) IMPC models against specific disease profiles, (C) MGI models scored against general disease profiles, and (D) MGI models scored against specific disease profiles. Sub-panels show the same data—one with a logarithmic vertical axis and one with a standard axis. (TIFF) [file pcbi.1007586.s015.tiff]

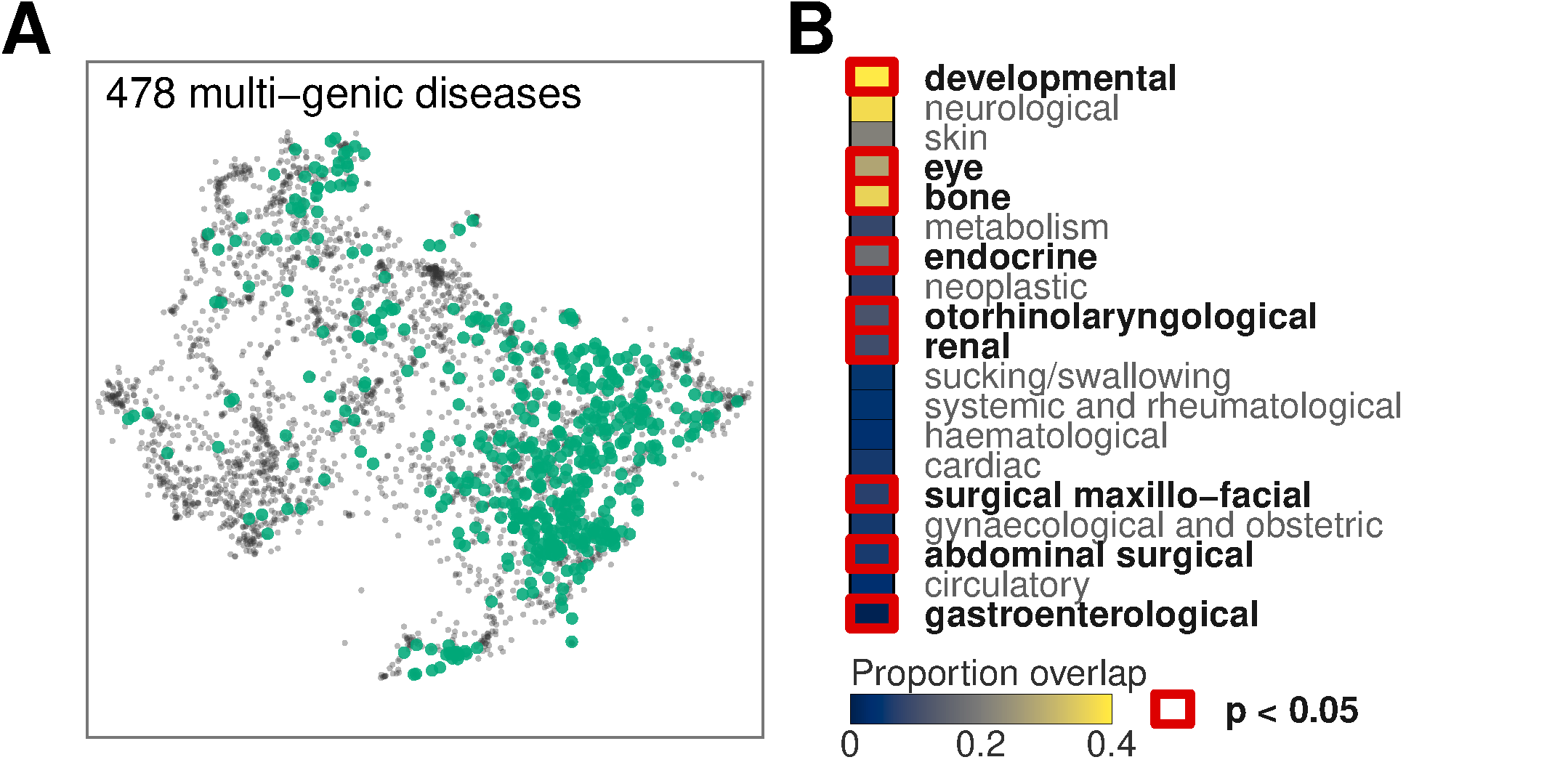

Supplement: S16 Fig — (A) Visualization of disease with many well-scored genes on the disease landscape map. The spread of highlighted dots on the map indicates that the disease set contains disease pertaining to a wide range of human pathology. (B) Statistical comparison of the multigenic disease set with curated disease categories. Heatmap boxes show overlap of the disease set with disease categories. Statistically unusual comparisons, evaluated using a Fisher test, are highlighted. (TIFF) [file pcbi.1007586.s016.tiff]

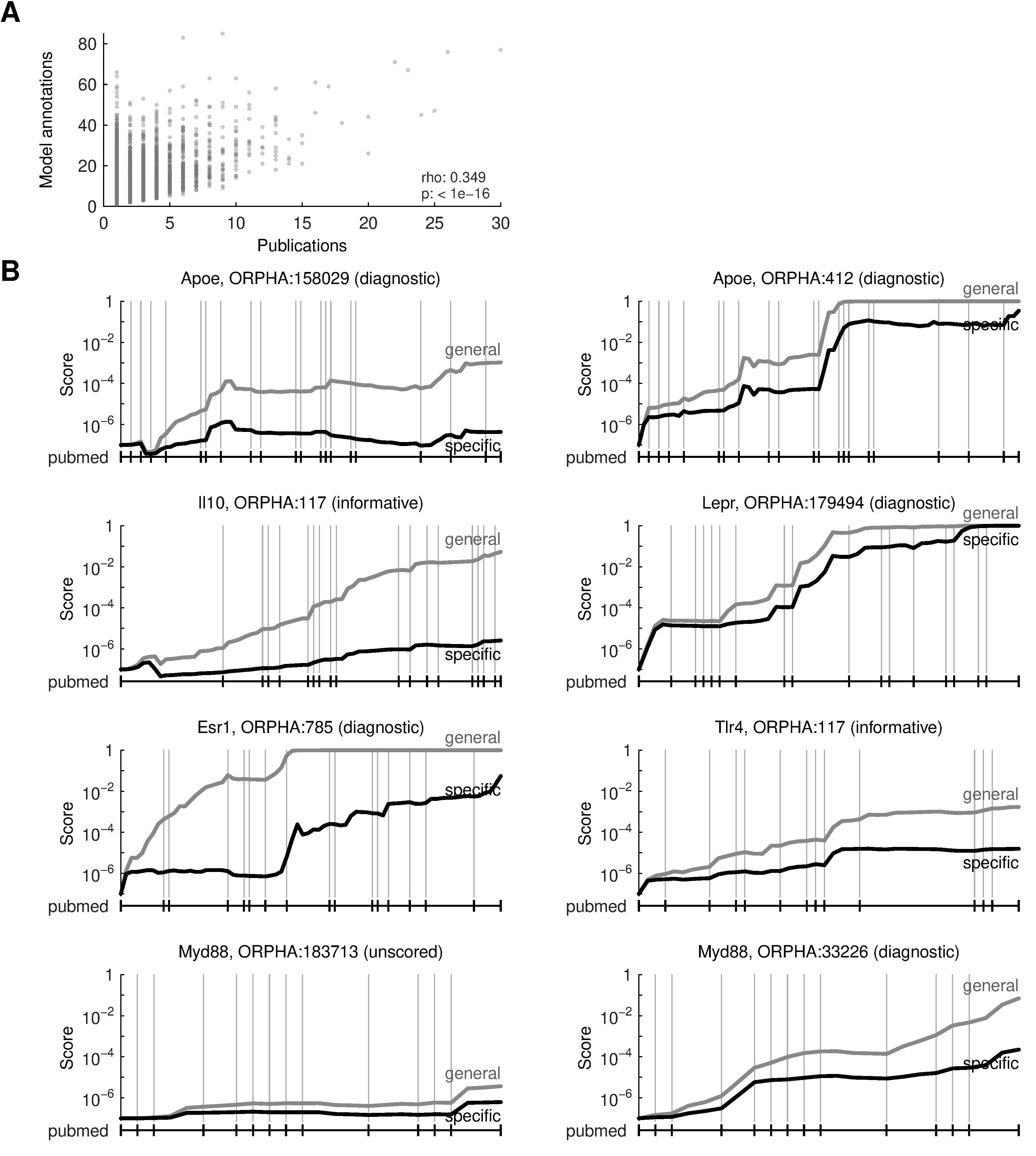

Supplement: S17 Fig — (A) Correlation between the number of publications associated with a model and the number of its distinct phenotypes. (B) Incremental scoring for models with the most publications and involving a known gene-disease associations. The horizontal axis tracks phenotypes, grouped by source publications (notches). Lines reveal scores against the general and specific profiles. Disease annotation quality is indicated in the titles. Interestingly, one panel shows that limited information about disease phenotypes limits mouse models to achieve high scores. (TIFF) [file pcbi.1007586.s017.tiff]

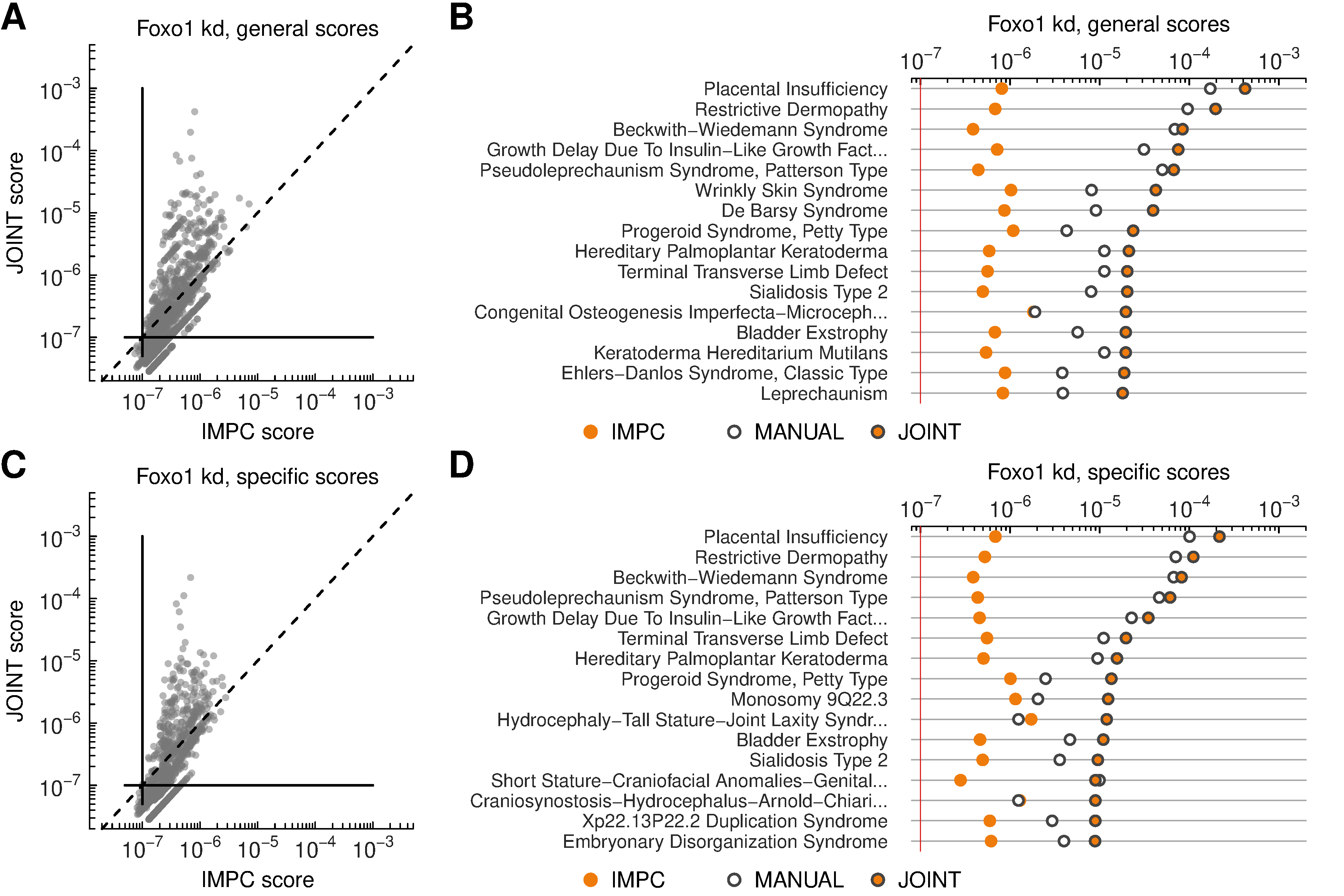

Supplement: S18 Fig — (A) Comparison of general scores computed for an IMPC model with Foxo1 knockout and a joint model consisting of IMPC and manually annotated phenotypes. (B) Top-ranking diseases for three related models involving gene Foxo1: phenotypes observed in an IMPC knock-out mouse (IMPC), manually curated phenotypes (MANUAL), and a joint model combining the IMPC and manual data (JOINT). (C) Analogous to (A), showing scores against specific disease profiles. (D) Analogous to (B), showing rankings against specific disease profiles. (TIFF) [file pcbi.1007586.s018.tiff]
